# miR-30a acts as a tumor suppressor by double-targeting COX-2 and BCL9 in *H. pylori* gastric cancer models

**DOI:** 10.1038/s41598-017-07193-w

**Published:** 2017-08-02

**Authors:** Xuan Liu, Qing Ji, Chengcheng Zhang, Xiaowei Liu, Yanna Liu, Ningning Liu, Hua Sui, Lihong Zhou, Songpo Wang, Qi Li

**Affiliations:** 1 Department of Medical Oncology, Shuguang Hospital, Shanghai University of Traditional Chinese Medicine, Shanghai, China; 2 Department of Medical Oncology, Longhua Hospital, Shanghai University of Traditional Chinese Medicine, Shanghai, China; 3 Department of Traditional Chinese Medicine, Shaanxi Provincial People’s Hospital, Shaanxi, China; 4 Department of Chinese Internal Medicine, Huzhou Hospital of Traditional Chinese Medicine, Zhejiang University of Traditional Chinese Medicine, Zhejiang, China; 5 Department of Traditional Chinese Medicine, Shanghai First People’s Hospital, Shanghai Jiaotong University School of Medicine, Shanghai, China

## Abstract

*Helicobacter pylori* (*H. pylori*) is one of the most important factors that affect the development of gastric cancer, and its mechanism remains un-elucidated. Our present study found that, miR-30a is crucial for regulating the growth and migration of *H. pylori* infected gastric cancer *in vitro* by targeting COX-2 and BCL9. In details, double-stranded miR-30a precursor produced two single-stranded and matured miRNAs including miR-30a-3p and miR-30a-5p, which played significant biological functions in two different manners. First, miR-30a-3p inhibited COX-2 expression and regulated nuclear translocation of β-catenin, and second, miR-30a-5p targeted BCL9 to regulate TCF/LEF promoter activity followed by affecting β-catenin downstream target gene expression. *In vivo*, miR-30a knockout mice were successfully achieved using CRISPR/Cas9 gene editing technology. Compared with *H. pylori*-infected wild-type mice, *H. pylori*-infected miR-30a knockout mice showed increased incidence of chronic gastritis, chronic atrophic gastritis, atypical hyperplasia, and other precancerous lesions or adenocarcinoma manifestations in the antral or gastric mucosa of mice, as well as regulation of genes closely associated with tumor development. Taken together, miR-30a acts as a tumor suppressor by double-targeting COX-2 and BCL9, and significantly affects the development of *H. pylori*-induced gastric cancer, shedding new light on the mechanisms underlying *H. pylori*-associated gastric cancer.

## Introduction

*Helicobacter pylori* (*H. pylori*) is an important pathogenic factor for gastric cancer^1–4^, and the International Agency for Research on Cancer (IARC) and World Health Organization (WHO) have attributed *H. pylori* as the first class carcinogenic factor for gastric cancer^5, 6^. Previous studies have shown that *H. pylori* can activate p38MAPK signaling pathway and thereby increase the expression of the COX-2 gene^7–9^. However, blocking the p38MAPK signaling pathway cannot completely reduce COX-2 expression. We speculate that there may be other ways to regulate COX-2 expression, and specifically if microRNAs (miRNAs) participate in the regulation^10, 11^. In the present study, our primary purpose was to determine the miRNAs that regulate COX-2 expression in *H. pylori*-infected gastric cancer cells, and to further explore the key miRNAs that regulate COX-2, as well as downstream gene expression or signaling pathways involved. miR-30a-3p was identified by microarray analysis as one of the upstream regulatory factors that target COX-2, and its regulatory mechanisms associated with the β-catenin signaling pathway^12, 13^ are the new findings to be elaborated in this study.

Bioinformatics analysis has shown that miR-30a-3p is just one strand of a double-stranded miR-30a precursor with a stem-loop structure, and our next analysis focused on the role that its complementary strand miR-30a-5p plays in *H. pylori*-infected gastric cancer cells. Sequence alignment results indicated that there are complementary binding sites between miR-30a-5p and the 3′UTR region of B-cell CLL/lymphoma 9 (BCL9) tumor suppressor gene^14, 15^. However, the detailed mechanism of miR-30a-5p targeting BCL9 to regulate the downstream gene expression or involved signaling pathways in *H. pylori*-infected gastric cancer is unclear. Therefore, another aim of the current study was to determine if miR-30a-5p targets BCL9 to regulate TCF/LEF promoter activity^16^ and downstream gene expression of β-catenin.

In addition, the biological function of miR-30a in the development of *H. pylori*-infected gastric cancer is also not fully understood. Our CRISPR/Cas9-mediated miR-30a knockout mice and previously well-built gastric cancer mouse models generated by *H. pylori* infection for long-time provide us the tools to elaborate the function of miR-30a *in vivo*. Therefore, in this study, we also showed the important roles of miR-30a in the development of *H. pylori*-induced gastric cancer.

## Results

### Screening and validation of the miRNAs regulating the COX-2 gene

**Figure 1:**
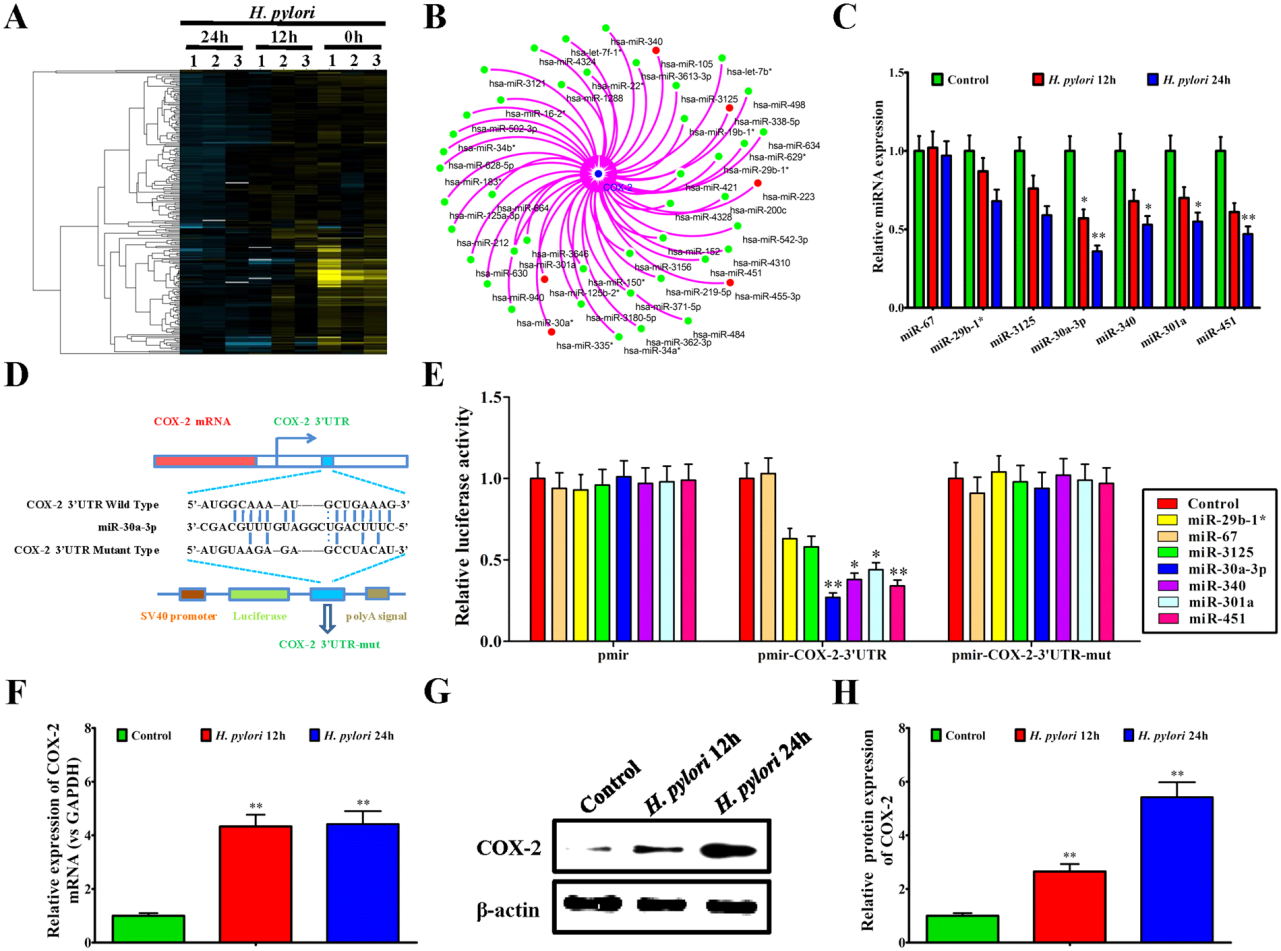
Screening and validation of the miRNAs regulating the COX-2 gene. (**A**) Cluster analysis of differentially expressed miRNAs in MKN45 cells and *H. pylori* infected MKN45 cells for 12 h and 24 h. Yellow color represents high expression and blue color represents low expression. The color brightness of each unit is associated with differences in multiples (log 2(AR/N). (**B**) Interaction network diagram between COX-2 and differentially expressed miRNAs using prediction software of microrna.org, mirDB, Targetscan 7.1 and starBase v2.0. (**C**) qRT-PCR assay of miR-29b-1^*^, miR-3125, miR-30a-3p, miR-340, miR-301a, and miR-451 in *H. pylori* infected MKN45 cells for 12 h and 24 h, normalized to miR-67 (irrelevant control). (**D**) Sequence alignment of the COX-2 3′UTR with wild-type (WT) versus mutant (mut) potential miR-30a-3p targeting sites. (**E**) Luciferase reporter activities of wild-type, mutant COX-2 3′UTR reporter, and empty constructs in HEK293T cells overexpressing miR-29b-1^*^, miR-67, miR-3125, miR-30a-3p, miR-340, miR-301a or miR-451, normalized to control HEK293T cells. (**F**) qRT-PCR assay of COX-2 mRNA in *H. pylori*-infected MKN45 cells for 12 h and 24 h, normalized to MKN45 cells without *H. pylori* infection. (**G**) Western blot assay of COX-2 protein in *H. pylori*-infected MKN45 cells for 12 h and 24 h. (**H**) Quantitative analysis of COX-2 protein in *H. pylori*-infected MKN45 cells for 12 h and 24 h, normalized to MKN45 cells without *H. pylori* infection. Data are shown as mean ± SD; n = 3. *p < 0.05, was considered as statistically significant, **p < 0.01, was considered as statistically highly significant.

Using microarray analysis, numerous abnormally expressed miRNAs were screened out from *H. pylori*-infected MKN45 cells, compared to *H. pylori*-uninfected MKN45 cells (Fig. 1A). Based on the microrna.org, mirDB, Targetscan 7.1 and starBase v2.0 network databases, we obtained a library of human miRNAs that target regulation of the COX-2 gene. By cross-checking, we constructed the interaction network diagram between the down-regulated miRNAs in *H. pylori*-infected MKN45 cells and the miRNAs that target the COX-2 gene (Fig. 1B). Further systematical analysis demonstrated that, miR-29b-1, miR-3125, miR-30a-3p, miR-340, miR-301a and miR-451 were the most significantly down-regulated miRNAs that target the COX-2 gene in *H. pylori*-infected MKN45 cells (Fig. 1A,B).

Next, real-time PCR was performed to further validate the differentially expressed miRNAs after *H. pylori* infection in MKN45 cells for 12 h and 24 h. The results showed that, all the miRNAs including miR-29b-1, miR-3125, miR-30a-3p, miR-340, miR-301a, and miR-451 showed decreased expression of varying levels, and the downregulation of miR-30a-3p expression was particularly notable (Fig. 1C). To verify the targeting effect, a dual luciferase reporter assay was applied to test the potential targeted regulation of each miRNA on the COX-2 3′UTR in HEK293T cells. Figure 1D showed a diagrammatic sketch of the specific binding between miR-30a-3p and the COX-2 3′UTR. It was found that, all the miRNAs have inhibitory effect on the dual luciferase activity of COX-2 3′UTR, and miR-30a-3p showed the most significant inhibitory effect (Fig. 1E). Furthermore, after *H. pylori* infection in MKN45 cells for 12 h and 24 h, both COX-2 mRNA and COX-2 protein expression were significantly upregulated (Fig. 1F,G,H).

### Inhibitory effect of miR-30a-3p on the growth and migration of *H. pylori*-infected gastric cancer cells

**Figure 2:**
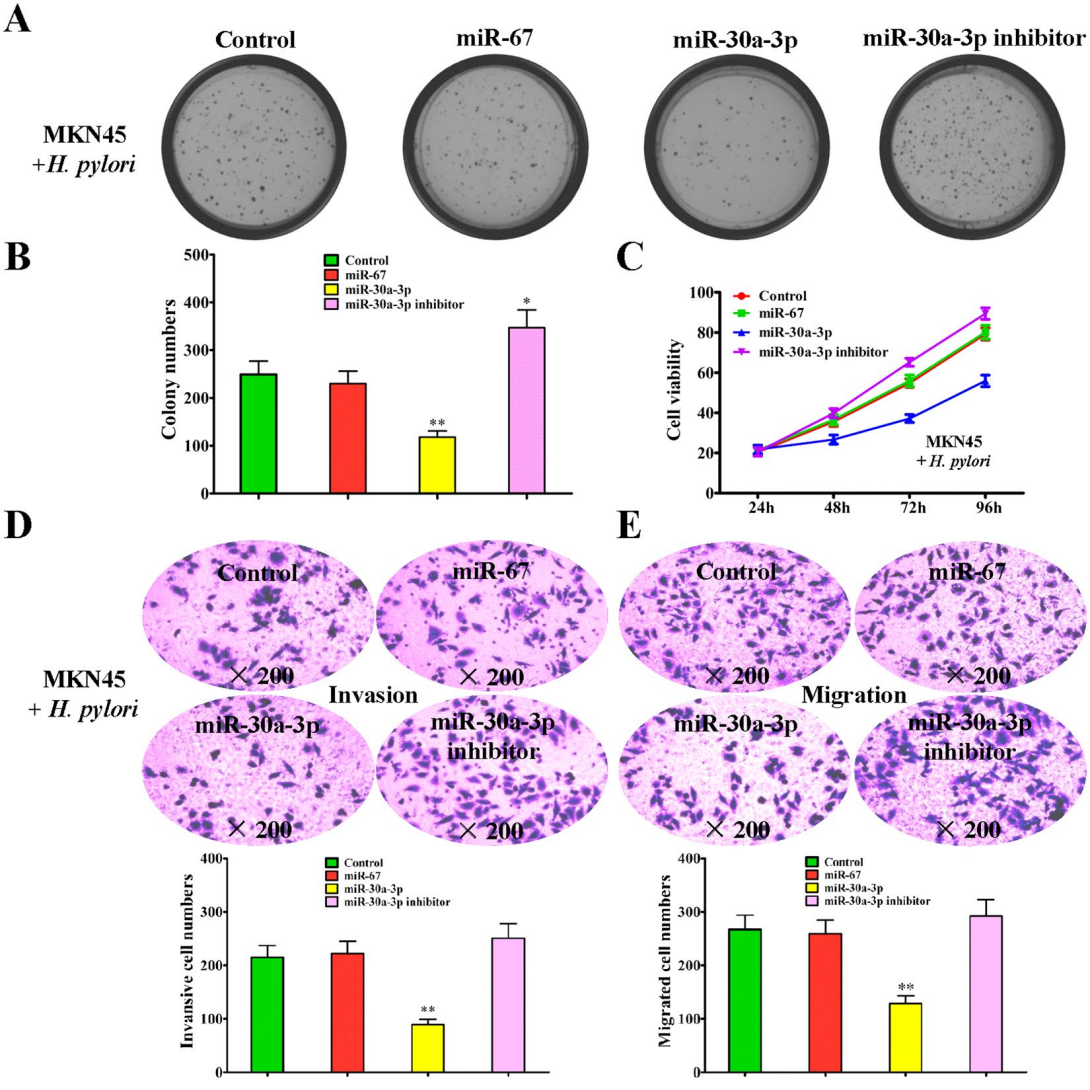
Regulatory effect of miR-30a-3p on the growth and migration of *H. pylori*-infected gastric cancer cells. (**A**) Colony formation assay of *H. pylori*-infected MKN45 cells transfected with miR-67 mimic, miR-30a-3p mimic, and miR-30a-3p inhibitor, respectively. *H. pylori*-infected MKN45 cells were used as a blank control. (**B**) Quantitative results of the colony formation numbers. (**C**) MTT assay of the cell viability of *H. pylori*-infected MKN45 cells transfected with miR-67 mimic, miR-30a-3p mimic and miR-30a-3p inhibitor, respectively. *H. pylori*-infected MKN45 cells were used as a blank control. (**D,E**) Invasion and migration assays of *H. pylori*-infected MKN45 cells transfected with miR-67 mimic, miR-30a-3p mimic and miR-30a-3p inhibitor, respectively. *H. pylori*-infected MKN45 cells were used as a blank control Numbers of invasive and migrated cells were shown as mean ± SD, n = 3. **p < 0.01 by Student’s t test. All the results were reproducible in three independent experiments.

Considering the importance of miR-30a-3p, we performed further investigation in *H. pylori*-infected MKN45 cells. In the preliminary experiments, we have found that, 12 h after *H. pylori* infection of MKN45 cells, the growth and colony formation of MKN45 cells increased significantly (Fig. 1A,B), as well as their invasion and migration abilities (Fig. 1C,D). As is shown in Fig. 2A–C, the miR-30a-3p mimic significantly inhibited the growth and colony formation of *H. pylori*-infected MKN45 cells, whereas the miR-30a-3p inhibitor blocked the inhibitory effect of *in situ* miR-30a-3p on the growth and colony formation of *H. pylori*-infected MKN45 cells. Similarly, transwell experiments also showed that, the miR-30a-3p mimic inhibited the invasion and migration of *H. pylori*-infected MKN45 cells, whereas the miR-30a-3p inhibitor blocked the inhibitory effect of *in situ* miR-30a-3p on the invasion and migration abilities of *H. pylori*-infected MKN45 cells (Fig. 2D,E).

### Regulatory effect of miR-30a-3p on the COX-2 expression and nuclear translocation of β-catenin in *H. pylori*-infected gastric cancer cells

**Figure 3:**
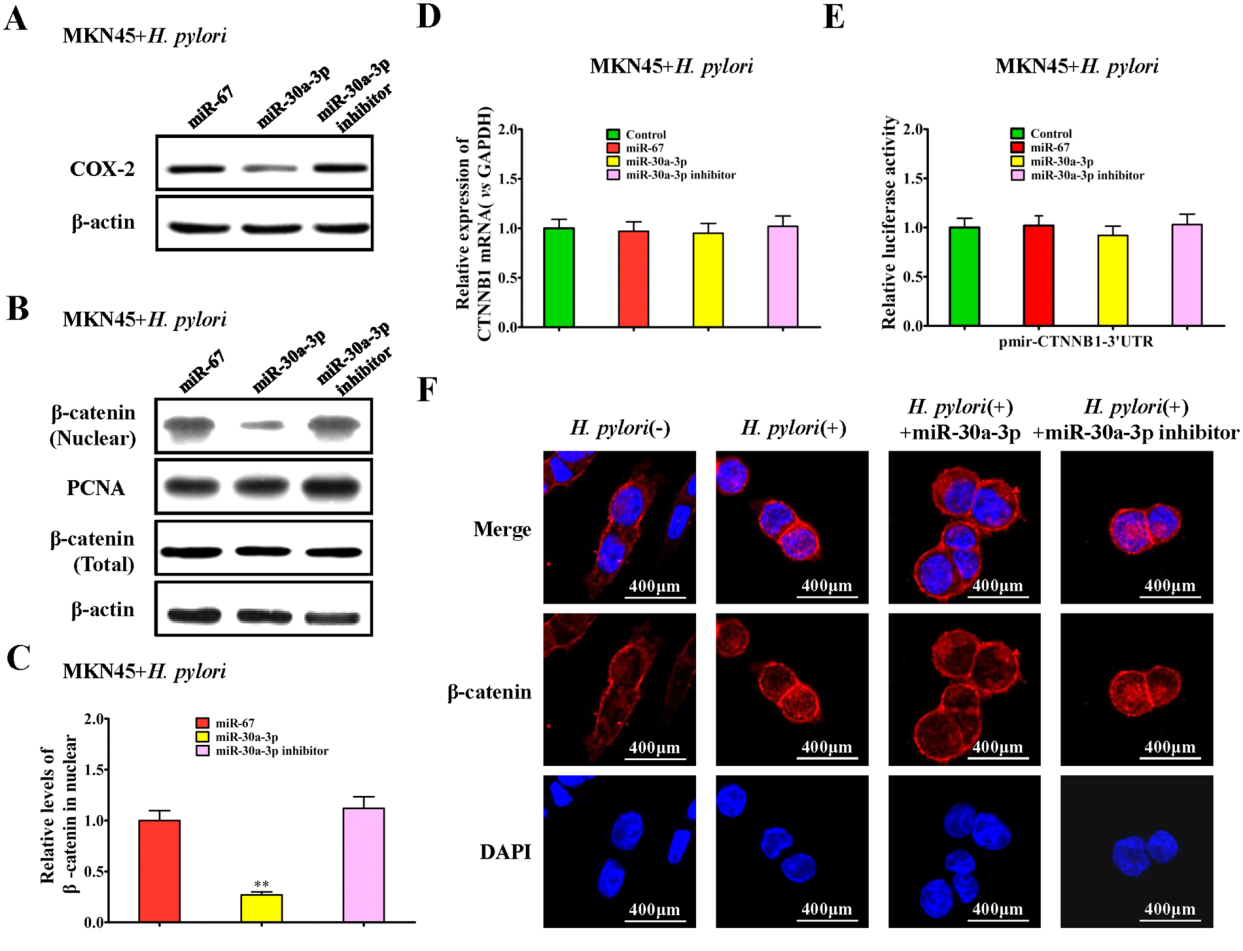
Regulation of miR-30a-3p on the COX-2 and β-catenin signaling pathway in *H. pylori*-infected gastric cancer cells. (**A**) Western blot assay for the effect of miR-30a-3p mimic and miR-30a-3p inhibitor on the COX-2 protein in *H. pylori*-infected MKN45 cells. (**B**,**C**) Western blot and quantitative assay for the effect of miR-30a-3p mimic and miR-30a-3p inhibitor on the nuclear translocation of β-catenin protein in *H. pylori*-infected MKN45 cells, normalized to *H. pylori*-infected MKN45 cells treated with miR-67 mimic. (**D**) qRT-PCR assay for the effect of miR-30a-3p mimic and miR-30a-3p inhibitor on β-catenin mRNA expression in *H. pylori*-infected MKN45 cells, normalized to control *H. pylori*-infected MKN45 cells. (**E**) Luciferase reporter activities assay for the effect of miR-30a-3p mimic and miR-30a-3p on the LEF/TCF promoter in *H. pylori*-infected MKN45 cells, normalized to control *H. pylori*-infected MKN45 cells. (**F**) Immunofluorescence β-catenin staining in *H. pylori* infected MKN45 cells transfected with miR-30a-3p mimic or miR-30a-3p inhibitor for 72 hours, and the *H. pylori*-infected MKN45 cells and *H. pylori*-uninfected MKN45 cells were the controls. Cell nuclei were stained with DAPI.

The preliminary results showed that miR-30a-3p regulated COX-2 mRNA expression but not COX-2 protein expression. Western blot analysis showed that, in *H. pylori*-infected MKN45 cells, the miR-30a-3p mimic significantly inhibited the expression of COX-2 protein, whereas miR-30a-3p inhibitor partly blocked the inhibitory effect of *in situ* miR-30a-3p on the expression of COX-2 protein (Fig. 3A).

Previous studies have suggested an impact of *H. pylori* on the β-catenin signaling pathway^9^, therefore, we next observed the regulatory effect of miR-30a-3p on the β-catenin involved signaling pathway. Our studies indicated that, in *H. pylori*-infected MKN45 cells, the miR-30a-3p mimic significantly inhibited nuclear translocation of β-catenin, while the miR-30a-3p inhibitor partly increased the nuclear translocation of β-catenin but without significant differences (Fig. 3B,C). However, the miR-30a-3p mimic had no direct effect on the mRNA and total protein expression of β-catenin (Fig. 3B–D), and miR-30a-3p also had little effect on the dual luciferase activity of the 3′UTR of β-catenin encoding gene (CTNNB1) (Fig. 3E). Immunofluorescence results also showed that, after *H. pylori* infection in MKN45 cells, the quantity of β-catenin protein in the nucleus was significantly increased. However, when the MKN45 cells were treated with the *H. pylori* and miR-30a-3p mimic simultaneously, the quantity of β-catenin protein in the nucleus significantly reduced, whereas the miR-30a-3p inhibitor had the opposite effect compared to the miR-30a-3p mimic (Fig. 3F). These results suggested that, miR-30a-3p could affect the biological function of *H. pylori*-infected gastric cancer cells by regulating COX-2 expression and nuclear translocation of β-catenin protein.

### Regulatory effect of miR-30a-5p on the BCL9-TCF/LEF signaling pathway in *H. pylori*-infected gastric cancer cells

Bioinformatics analysis indicated that, miR-30a-5p, another production of the miR-30a precursor, showed targeted binding sites on the 3′UTR of the BCL9 gene, which is the critical auxiliary protein in the β-catenin signaling pathway.

**Figure 4:**
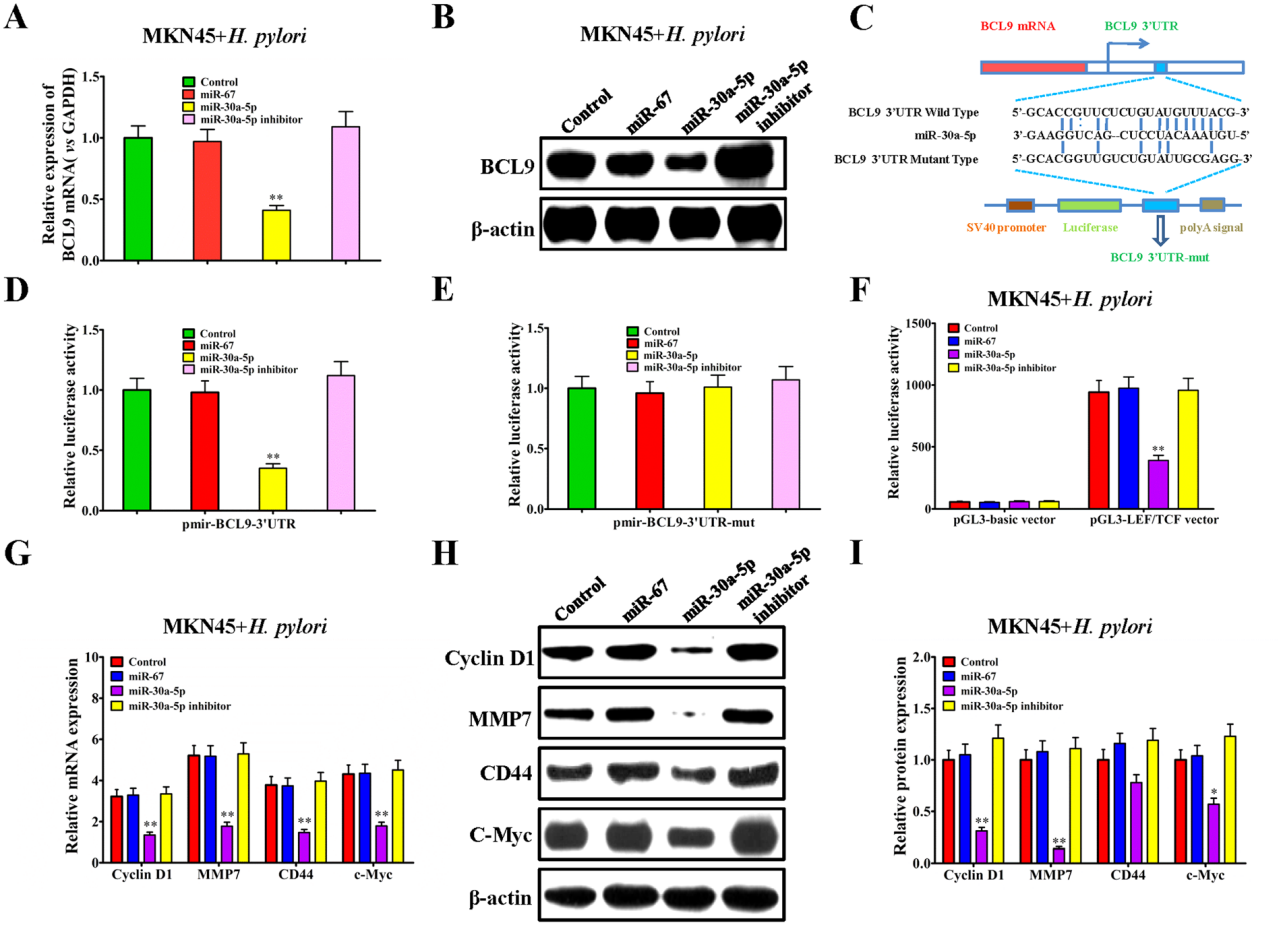
Regulation of miR-30a-5p on the BCL9-TCF/LEF signaling pathway in *H. pylori*-infected gastric cancer cells. (**A**) qRT-PCR assay for the effect of miR-30a-5p mimic and miR-30a-5p inhibitor on BCL9 mRNA expression in *H. pylori*-infected MKN45 cells, normalized to control *H. pylori*-infected MKN45 cells. (**B**) Western blot assay for the effect of miR-30a-5p mimic and miR-30a-5p inhibitor on BCL9 protein expression in *H. pylori*-infected MKN45 cells. (**C**) Sequence alignment of the BCL9 3′UTR with wild-type (WT) versus mutant (mut) potential miR-30a-5p targeting sites. (**D**,**E**) Luciferase reporter activity assay of wild-type, mutant BCL9 3′UTR reporter in MKN45 cells overexpressing miR-30a-5p in HEK293T cells, normalized to control *H. pylori*-infected MKN45 cells. (**F**) Luciferase reporter activity assay for the effect of miR-30a-5p mimic and miR-30a-5p inhibitor on the LEF/TCF promoter in *H. pylori*-infected MKN45 cells, normalized to control *H. pylori*-infected MKN45 cells. (**G**) qRT-PCR assay for the effect of miR-30a-5p mimic and miR-30a-5p inhibitor on the transcriptional activity of β-catenin downstream target genes including Cyclin D1, MMP7, CD44 and c-Myc in *H. pylori*-infected MKN45 cells, normalized to control *H. pylori*-infected MKN45 cells. (**H**), (**I**) Western blot and quantitative assay for the effect of miR-30a-5p mimic and miR-30a-5p inhibitor on the protein expression of BCL9 and β-catenin downstream target genes including Cyclin D1, MMP7, CD44 and c-Myc in *H. pylori*-infected MKN45 cells, normalized to control *H. pylori*-infected MKN45 cells. Data are shown as mean ± SD; n = 3. *p < 0.05, was considered as statistically significant, **p < 0.01, was considered as statistically highly significant.

In preliminary experiments, we demonstrated that, after *H. pylori* infection in MKN45 cells for 12 h and 24 h, miR-30a-5p showed significantly decreased expression (Fig. 2A). Moreover, the mRNA and protein expression of the BCL9 gene in MKN45 cells also increased significantly after *H. pylori* infection in MKN45 cells for 24 h (Fig. 2B, C). Next, we further investigated the effect of miR-30a-5p on the BCL9-TCF/LEF signaling pathway in *H. pylori*-infected gastric cancer MKN45 cells. Real-time PCR results indicated that, in *H. pylori*-infected MKN45 cells, the miR-30a-5p mimic significantly inhibited the expression of BCL9 mRNA, which was consistent with the results of the dual luciferase detection (Fig. 4A). Further studies showed that, in *H. pylori*-infected MKN45 cells, BCL9 protein expression was significantly increased, and the miR-30a-5p mimic could significantly reduce the expression of BCL9 compared to the miR-67 mimic, while the miR-30a-5p inhibitor blocked the inhibitory effect of *in situ* miR-30a-5p on BCL9 protein expression (Fig. 4B). Targeting the binding sites, we designed the dual luciferase reporter vector pmirGLO-BCL9-3′UTR, and the pmirGLO-BCL9-3′UTR-mut vector, which was mutated in the binding sites between miR-30a-5p and the 3′UTR of the BCL9 gene (Fig. 4C). The pmirGLO-BCL9-3′UTR plasmid (or pmirGLO-BCL9-3′UTR-mut, or pmirGLO) and miR-30a-5p mimic (or miR-67 mimic, or miR-30a-5p inhibitor) were co-transfected into HEK293T cells. After 48 h, the luciferase activities were detected. For pmirGLO-BCL9-3′UTR, compared with the miR-67 mimic group, the miR-30a-5p mimic group showed decreased luciferase activity, whereas the miR-30a-5p inhibitor group showed significantly increased luciferase activity. Compared with the pmirGLO-BCL9-3′UTR, the miR-30a-5p mimic had little effect on the luciferase activity of pmirGLO-BCL9-3′UTR-mut (Fig. 4D,E). These results suggested that miR-30a-5p can significantly inhibit the expression of BCL9.

BCL9 protein is the critical auxiliary protein to activate Wnt/β-catenin signaling pathway, and is essential for downstream target gene transcription. In the nucleus, cofactors such as BCL9, Pygo, β-catenin and transcription factor TCF/LEF interact with each other and bind together to the genomic DNA, and turn on the transcription of downstream target genes such as Cyclin D1, MMP7, CD44 and c-Myc. Accordingly, we tested the effect of miR-30a-5p on the promoter activity of TCF/LEF. In preliminary experiments, we found that, after *H. pylori* infection in MKN45 cells for 24 h, the dual luciferase activity of the TCF/LEF promoter was significantly up-regulated (Fig. 2D). In *H. pylori*-infected MKN45 cells, *H. pylori* significantly increased the dual luciferase activity of the TCF/LEF promoter, and the miR-30a-5p mimic significantly decreased the dual luciferase activity of the TCF/LEF promoter compared with the miR-67 mimic group, while the miR-30a-5p inhibitor blocked the inhibitory effect of *in situ* miR-30a-5p on the dual luciferase activity of the TCF/LEF promoter but there was no statistically significant difference between the effects of the miR-67 mimic and miR-30a-5p inhibitor (Fig. 4F). These results showed that, miR-30a-5p can significantly reduce the promoter activity of TCF/LEF.

Previously, we have showed that *H. pylori* infection can significantly up-regulated the expression of β-catenin downstream target genes such as Cyclin D1, MMP7, CD44 and c-Myc (Fig. 2E,F). In this study, the results demonstrated that, in *H. pylori*-infected MKN45 cells, compared with the miR-67 mimic, the miR-30a-5p mimic can significantly down-regulate the mRNA and protein expression of Cyclin D1, MMP7, CD44 and c-Myc, while the miR-30a-5p inhibitor can up-regulate the expression of β-catenin downstream target genes, but no statistical significance was found between the miR-30a-5p inhibitor and miR-67 mimic (Fig. 4G–I). These results indicated that, miR-30a-5p can target BCL9 to inhibit the promoter activity of TCF/LEF and transcriptional activity of β-catenin downstream target genes.

### miR-30a precursor targets COX-2 and BCL9 to regulate the growth and migration of *H. pylori*-infected gastric cancer cells

**Figure 5:**
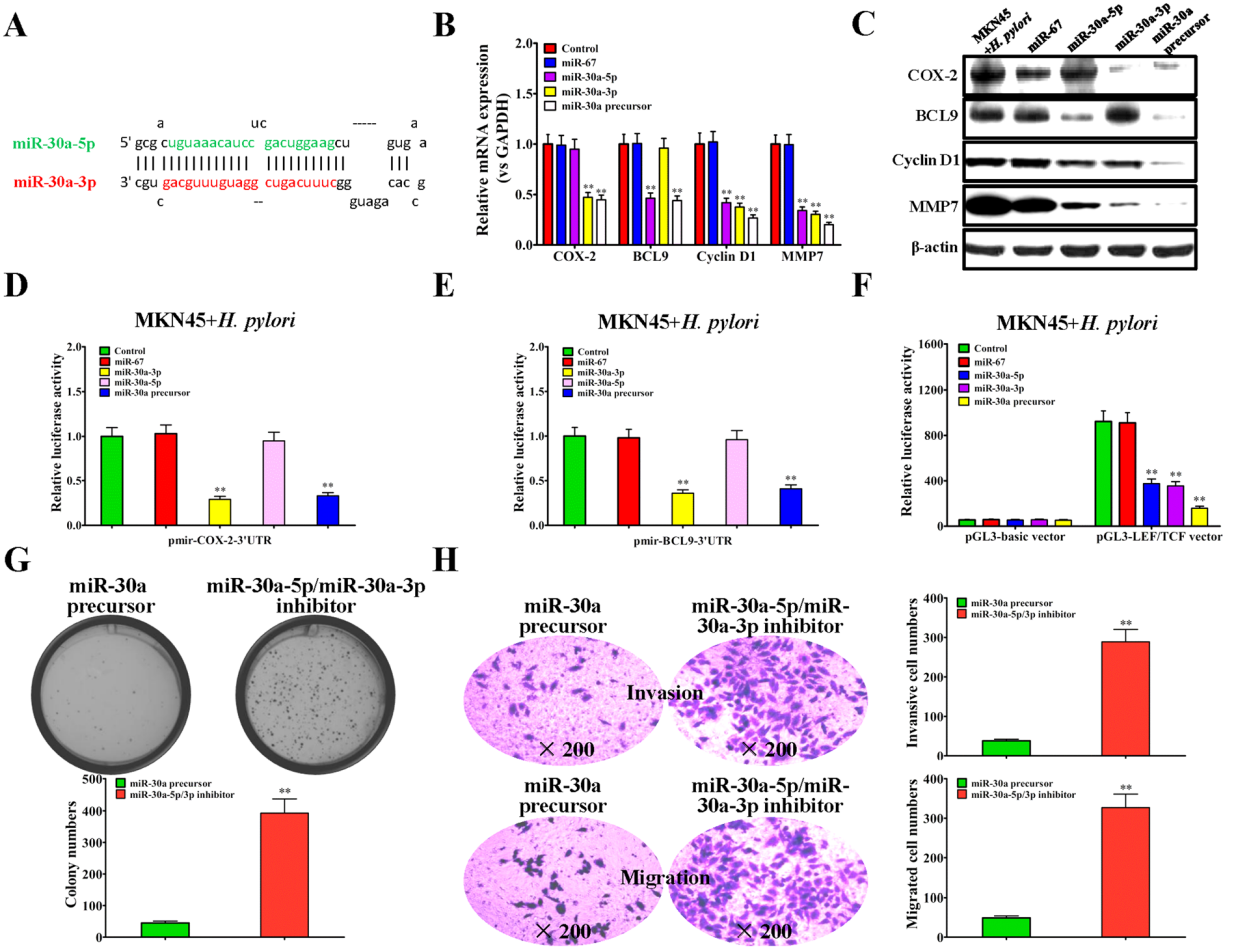
miR-30a precursor targets COX-2 and BCL9 to regulate the growth and migration of *H. pylori* infected gastric cancer cells. (**A**) Complementary stem-loop structure of the miR-30a precursor. (**B**) qRT-PCR assay for the effect of the miR-30a precursor on the transcriptional activity of COX-2, BCL9, Cyclin D1 and MMP7 in *H. pylori*-infected MKN45 cells, normalized to control *H. pylori*-infected MKN45 cells. (**C**) Western blot assay for the effect of miR-30a precursor on the protein expression of COX-2, BCL9, CyclinD1 and MMP7 in *H. pylori*-infected MKN45 cells. (**D**) Luciferase reporter activity assay for the effect of miR-30a precursor on the wild-type COX-2 3′UTR reporter, normalized to control *H. pylori*-infected MKN45 cells. (**E**) Luciferase reporter activity assay for the effect of the miR-30a precursor on the wild-type BCL9 3′UTR reporter, normalized to control *H. pylori*-infected MKN45 cells. (**F**) Luciferase reporter activities assay for the effect of miR-30a precursor on the LEF/TCF promoter in *H. pylori*-infected MKN45 cells, normalized to control *H. pylori*-infected MKN45 cells. (**G**) Colony formation assay of *H. pylori*-infected MKN45 cells transfected with the miR-30a precursor and miR-30a-5p/miR-30a-3p inhibitor (up), and the quantitative results of the colony formation numbers (down). (**H**) Invasion and migration assays of *H. pylori*-infected MKN45 cells transfected with the miR-30a precursor and miR-30a-5p/miR-30a-3p inhibitor (up), and the numbers of invasive and migrated cells were shown as mean ± SD, n = 3 (down). **p < 0.01 by Student’s t test. All the results were reproducible in three independent experiments.

miR-30a-3p and miR-30a-5p exhibit a complementary stem-loop structure in the presence of the miR-30a precursor. *In vivo*, the miR-30a precursor produces two different short miRNAs, accompanied by two different targets and pathways. Thus, we directly chemically synthesized the double-stranded miR-30a precursor for experimental verification (Fig. 5A). Compared with the individual miR-30a-3p or miR-30a-5p, the miR-30a precursor had excellent inhibitory effects on both mRNA and protein expression of COX-2 and BCL9 in *H. pylori*-infected MKN45 cells (Fig. 5B,C), and the miR-30a precursor also showed a double inhibitory effect on the luciferase activities of pmirGLO-COX-2-3′UTR and pmirGLO-BCL9-3′UTR (Fig. 5D). Moreover, in *H. pylori*-infected MKN45 cells, compared with the miR-30a-3p mimic or miR-30a-5p mimic, the miR-30a precursor showed an increased inhibitory effect on the LEF/TCF promoter activity (Fig. 5E) and the expression of β-catenin downstream target genes such as Cyclin D1 and MMP7 (Fig. 5B,C). In addition, in compared with the miR-30a-3p mimic, the miR-30a precursor had an increased effect on the growth and migration of *H. pylori*-infected MKN45 cells (Figs 2D,E and 5G,H).

In addition, the above molecular mechanisms were validated in SGC-7901 gastric cancer cells. The results demonstrated that, *H. pylori* infection significantly up-regulated the expression of COX-2, BCL9, Cyclin D1 and MMP7 in SGC-7901 cells (Fig. 3A,B), and promoted the LEF/TCF promoter activity (Fig. 3C). However, as observed in MKN45 cells, compared with individual miR-30a-3p or miR-30a-5p, the miR-30a precursor had an increased inhibitory effect on COX-2, BCL9, Cyclin D1 and MMP7 expression in *H. pylori*-infected SGC-7901 cells (Fig. 3D,E), and an increased inhibitory effect on LEF/TCF promoter activity (Fig. 3F). Additionally, *H. pylori* infection also significantly promoted the growth, invasion and migartion ability of SGC-7901 cells (Fig. 4A,B). Similarly, the miR-30a precursor had very pronounced inhibitory effect on the growth and migration of *H. pylori*-infected SGC-7901 cells (Fig. 4C,D). All the above results demonstrated that, the miR-30a precursor targets COX-2 and BCL9 to regulate the growth and migration of *H. pylori*-infected gastric cancer cells.

### Construction of miR-30a knockout mouse model using CRISPR/Cas9 technology

**Figure 6:**
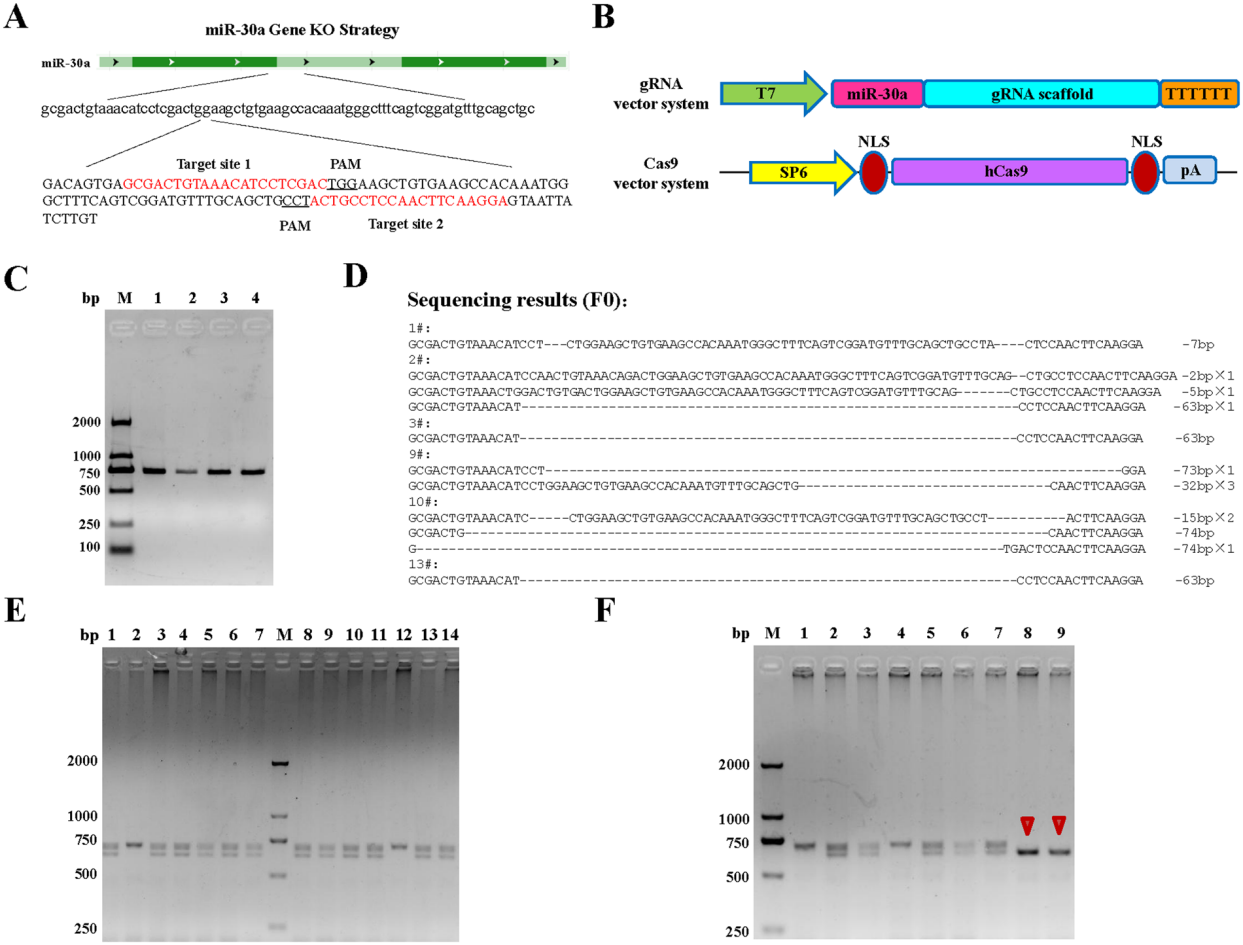
Construction of miR-30a knockout mouse model using CRISPR/Cas9 technology. (**A**) Two CRISPR nuclease gRNAs were designed for miR-30a knockout. (**B**) Schematic diagram of the gRNA vector system and Cas9 vector system. (**C**) *In vitro* transcription template (716 bp DNA fragment) was obtained by PCR amplification and characterized by gel electrophoresis and DNA sequencing. (**D**) Validation of CRISPR miR-30a knockout in F0 generation mice by PCR and DNA sequencing. (**E**) miR-30a heterozygous F1 generation mice were screened out by PCR amplification from the 14 posterity of F0 generation mice mating with wild-type mice. (**F**) Two miR-30a gene knockout homozygous mice (marked with red arrow) were identified from the 9 posterity of two-two mating of heterozygous F1 generation mice.

Using CRISPR/Cas9 gene targeting techniques, gRNA targeting miR-30a was built, and used to guild Cas9 protein to shear DNA duplexes at specific sites. Since miR-30a is a short, non-coding sequence, the appropriate target was chosen for gRNA designing. In addition, considering target efficiency and identification, double targets were designed (Fig. 6A). A schematic diagram of the gRNA vector system and Cas9 vector system are presented in Fig. 6B. By PCR amplification and gel electrophoresis, a DNA fragment of the expected size (716 bp) was obtained and confirmed by sequencing (Fig. 6C).

The gRNA targeting miR-30a and the Cas9 mRNA were *in vitro* transcribed respectively, mixed and diluted for microinjection. Immediately after injection, the zygote was transplanted into pseudopregnant C57BL/6 female mice. The genomic DNAs of the newborn mice were extracted for PCR amplification and identification by DNA sequencing. The results showed that, each mouse had varying degrees of base insertion or deletion mutations in the protospacer adjacent motif (PAM) sequence, wherein the first-building mice of No. 9, No. 10, and No. 13 produced more than 60 missing bases, indicating that miR-30a had achieved efficient knockout in mice (Fig. 6D).

The above-identified F0 generation mice were mated with wild-type mice, and 14 F1 generation mice were produced. Twelve miR-30a heterozygous mice were screened out by PCR amplification from F1 generation mice (Fig. 6E). By two-two mating of heterozygous F1 generation mice, 9 F2 generation mice were produced. The produced mice of No. 8 and No. 9 were identified as miR-30a gene knockout homozygous mice (Fig. 6F), which could be used in the subsequent *H. pylori* infection experiments. In addition, the phenotype observation showed that, miR-30a knockout had little influence on the growth and development of the miR-30a knockout mice.

### *H. pylori*-infected miR-30a knockout mice show increased incidence of precancerous lesions and adenocarcinoma manifestations

**Figure 7:**
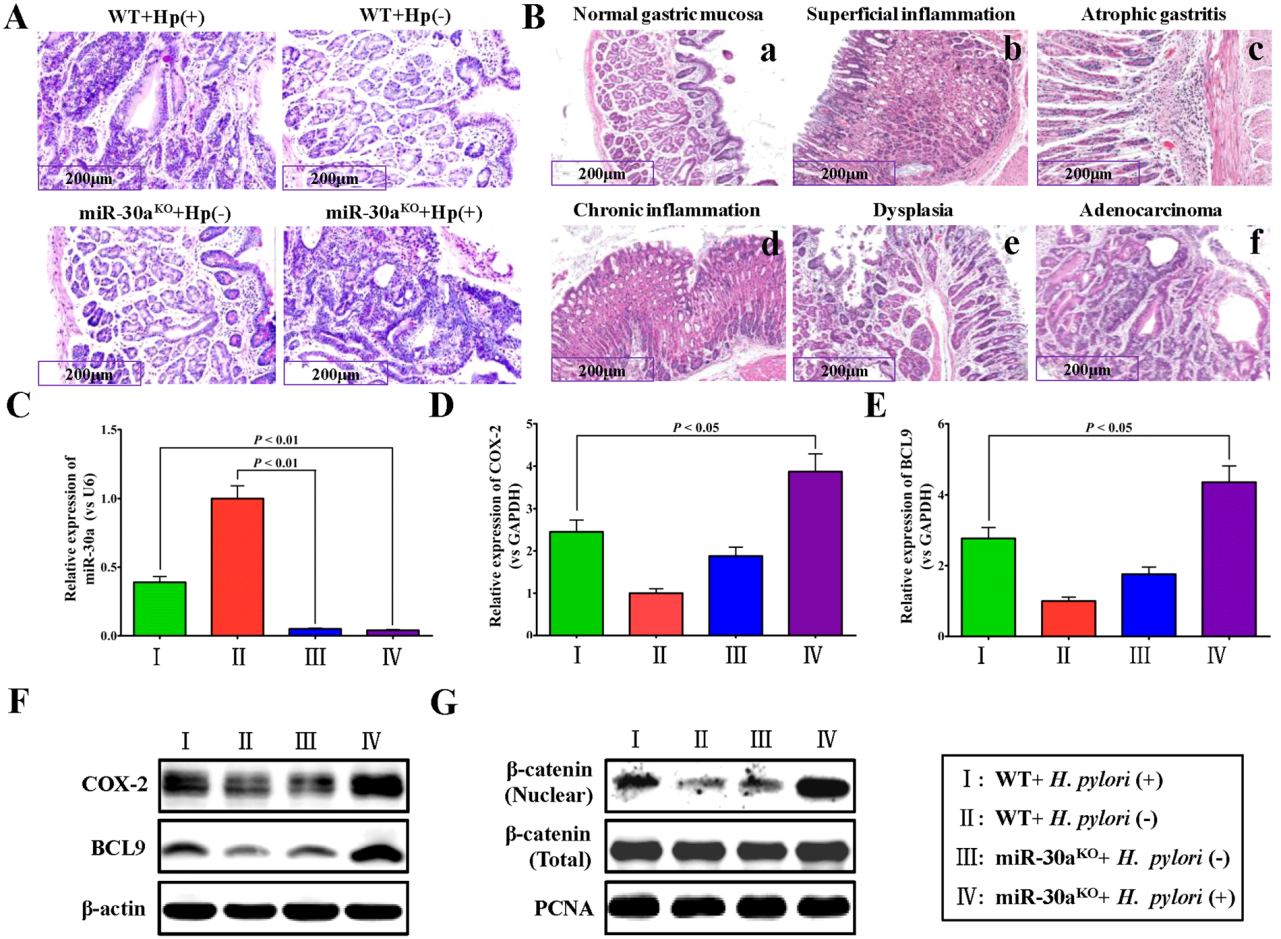
Expression of miR-30a, COX-2, BCL9, β-catenin, VEGF and CD34 in the *H. pylori*-infected miR-30a knockout mouse model. (**A**) Representative Giemsa staining results of the gastric mucosa from the miR-30a knockout (miR-30a^KO^) mice or wild-type (WT) mice infected by *H. pylori*. miR-30a knockout reduced both the expression levels of miR-30a-3p and miR-30-5p for their complementary stem-loop structure in the presence of miR-30a precursor. (**B**) Representative HE staining results of the gastric mucosa from the miR-30a^KO^ mice (d,e,f) or WT mice (b,c) infected by *H. pylori* or WT mice without *H. pylori* infection (a). (**C**) qRT-PCR assay for miR-30a in gastric mucosa from the miR-30a^KO^ mice or WT mice infected by *H. pylori*, normalized to WT mice without *H. pylori* infection. (**D**,**E**) qRT-PCR assay for COX-2 and BCL9 in gastric mucosa from the miR-30a^KO^ mice or WT mice infected by *H. pylori*, normalized to WT mice without *H. pylori* infection Data are shown as mean ± SD; n = 3. **p < 0.01, was considered as statistically highly significant. (**F**,**G**) Western blot and quantitative analysis for COX-2, BCL9 and β-catenin in gastric mucosa from the miR-30a^KO^ mice or WT mice infected by *H. pylori*. PCNA (Nuclear) and β-actin (Total) was used as a loading control.

The *in vivo* experiment demonstrated that, after *H. pylori* infection for 10 weeks, 25 weeks, and 45 weeks, whether in the control wild-type mouse model or in miR-30a knockout mouse model, the *H. pylori* colonization rates in the antrum and gastric mucosal were both very high, and at 45 weeks the *H. pylori* colonization rate of all the mice reached 100%, although several mice died of natural causes without obvious disease symptom by pathological examination. These results implied that, miR-30a knockout had little effect on the *H. pylori* colonization rate of mice (Fig. 7A, Table 1).

**Table 1:** Rapid urease test and Giemsa staining results for the gastric mucosa of the *H. pylori*-infected wild type (WT) mice and miR-30a^KO^ mice.

Group	0 weeks	10 weeks	25 weeks	45 weeks
	n (*H. pylori* ^+^)	n (*H. pylori* ^+^)	n (*H. pylori* ^+^)	n (*H. pylori* ^+^)
WT + *H. pylori*	20	19 ± 1	18 ± 2	18 ± 1
miR-30a^KO + ^ *H. pylori*	20	18 ± 2	18 ± 1	17 ± 1

Note: All the animal experiments were independently repeated three times, and the interval time between each time of experiment was about one month. In each experiment, the initial numbers of mice in each group for each time was 20. *H. pylori* ^+^ represents *H. pylori* positive. After 10 weeks, 25 weeks and 45 weeks, several of the 20 WT/*H. pylori* mice or the 20 miR-30a^KO^/*H. pylori* mice died of natural causes. The rapid urease test results were consistent with the Giemsa staining results.

After *H. pylori* infection for 72 weeks in the control wild-type mice model, 12 of 20 mice showed ulcers, mucosal surface roughness, and white and tip size particles scattered in the antrum, gastric body and their junction. More than 15 of 20 mice in the miR-30a knockout mouse model showed the pathological phenomena described above, but with more instances of focus and more serious pathological degree. Moreover, compared with the control wild-type mice model, 72 weeks after *H. pylori* infection, the miR-30a knockout mouse model displayed more chronic atrophic gastritis and dysplasia in the antral mucosa, and increased shrinking, dysplasia, and adenocarcinoma in the gastric mucosa (Fig. 7B, Table 2).

**Table 2:** Pathological examination for the gastric mucosa of the *H. pylori*-infected and -uninfected wild type (WT) mice or miR-30a^KO^ mice (72 weeks, HE staining).

Group	Superficial inflammation	Chronic inflammation	Atrophic gastritis	Dysplasia	Adenocarcinoma
	n	n	n	n	n
WT + *H. pylori*	17 ± 2	15 ± 1	11 ± 2	7 ± 1	2 ± 1
miR-30a^KO + ^ *H. pylori*	17 ± 1	16 ± 2	14 ± 2	11 ± 3	6 ± 2
WT	0	0	0	0	0
miR-30a^KO^	0	0	0	0	0

Note: All the animal experiments were independently repeated three times, and the interval time between each time of experiment was about one month. In each experiment, the initial numbers of mice in each group for each time was 20. After 72 weeks, several of the 20 WT/*H. pylori* mice or the 20 miR-30a^KO^/*H. pylori* mice died of natural causes.

Comprehensive analysis demonstrated that, both of the control wild-type mice model and miR-30a knockout mouse model showed chronic gastritis, chronic atrophic gastritis, dysplasia and adenocarcinoma in the gastric mucosa, and *H. pylori*-infected miR-30a knockout mice showed increased incidence of precancerous lesions and adenocarcinoma manifestations compared to the *H. pylori*-infected wild-type mice.

### Expression of COX-2, BCL9, β-catenin, VEGF and CD34 in the miR-30a knockout mouse model

Real-time PCR showed that, 72 weeks after *H. pylori* infection, compared with the *H. pylori*-infected wild-type mouse model, miR-30a expression in *H. pylori*-infected miR-30a knockout mouse model was significantly decreased (Fig. 7C). Meanwhile, the mRNA expression levels of both COX-2 (PTGS2) and BCL9, significantly increased in the *H. pylori*-infected miR-30a knockout mouse model comparing with the *H. pylori* infected wild-type mice (Fig. 7D,E).

Moreover, the immunohistochemistry (IHC) results showed that, 72 weeks after *H. pylori* infection, compared with the *H. pylori*-infected wild-type mouse model, the *H. pylori*-infected miR-30a knockout mouse model displayed significantly increased CD34 expression, as well as the COX-2 and VEGF expression (Fig. 5A,B). Western blot analysis of randomly selected tissues also showed that, the expression levels of COX-2 and BCL9 protein were significantly up-regulated in *H. pylori*-infected miR-30a knockout mouse model compared with the *H. pylori*-infected wild-type mice model (Fig. 7F). Additionally, the nuclear quantities of β-catenin protein significantly increased in the *H. pylori*-infected miR-30a knockout mouse model compared with the *H. pylori*-infected wild-type mouse model (Fig. 7G). All these results suggested that, miR-30a was associated closely with the development of *H. pylori*-induced gastric cancer, and regulated the genes closely associated with tumor development such as COX-2, BCL9, nuclear β-catenin, VEGF and CD34.

### Expression of COX-2, BCL9, VEGF and CD34 in human tissue samples

Finally, we evaluated the gene expression levels in patients and determined if they corresponded to our findings *in vitro* and in mouse models. Using IHC staining, we detected the protein expression levels of COX-2, BCL9, VEGF, and CD34 in tissue samples from patients with *H. pylori* gastritis, *H. pylori*-related gastric cancer and healthy controls. Compared with the healthy controls, higher expression levels of COX-2, BCL9, VEGF, and CD34 were observed in tissue samples from *H. pylori* gastritis and *H. pylori*-related gastric cancer, and the expression levels of these proteins were even higher in *H. pylori*-related gastric cancer compared to *H. pylori* gastritis (Figs 6 and 7A–D). In addition, we also found that, compared with the healthy controls, the expression levels of both miR-30a-5p and miR-30a-3p were down-regulated in the tissue samples from *H. pylori* gastritis and *H. pylori*-related gastric cancer, and the expression levels of miR-30a-5p and miR-30a-3p were even lower in *H. pylori*-related gastric cancer than that of *H. pylori* gastritis (Fig. 7E). All these results are in agreement with our findings in cell lines and mouse models.

## Discussion

Numerous researchers have found that, over-expression of COX-2 not only allows tumor cells to escape apoptosis and causes abnormal connections between cells to obtain an invasive phenotype, but it also increases angiogenesis production adjacent to tumor cells and promotes tumor angiogenesis^17–19^. A great number of factors can lead to the over-expression of COX-2 gene^20, 21^. Our previous study found that, *H. pylori*, a gram-negative bacterium, can activate the p38MAPK signaling pathway and increase COX-2 expression, but blocking the p38MAPK signaling pathway cannot completely reduce COX-2 expression^7–9^. Therefore, we hypothesized that there must be other factors or signaling pathways involved in regulating COX-2 expression.

In recent years, miRNAs have become of great interest to researchers. Generally, in the development of tumors, miRNAs play two roles as oncogenes or tumor suppressor genes. Oncogenic miRNAs often function as oncogenes, which are often in a state of high expression or persistent activation in tumor tissues^22^. Tumor suppressor miRNAs often play tumor suppressing effect, which are often low expressed or in inactivated state in tumor tissues^23^. In this study, using microarray analysis, bioinformatics prediction, dual luciferase activity experiments and other means, miR-30a-3p was identified to have an inhibitory effect on growth and migration of *H. pylori*-infected gastric cancer cells through targeting the COX-2 gene. Further experiments showed the important effect of miR-30a-5p on β-catenin associated LEF/TCF promoter activity and downstream target genes transcription such as Cyclin D1, MMP7, CD44 and c-Myc. Numerous studies have shown that, aberrant activation of the Wnt/β-catenin signaling pathway is involved in the pathogenesis of many human malignant tumors, and abnormal activation is due to the dysregulation of the Wnt gene or other member of the signaling pathway, such as β-catenin and GSK-3β^24, 25^.

In the bioinformatics screen for miR-30a targets, we found that miR-30a-5p, the reverse complementary sequence of miR-30a-3p, targeted binding sites of the key cofactor BCL9 in the Wnt/β-catenin signaling pathway. The BCL9 gene, named for its identification in a patients suffering from pre-B cell acute lymphoblastic leukemia (ALL), is located on chromosome 1q21 and often occurs in (1; 14) (q21; q23) chromosome translocation^14, 15^. BCL9 is a newly discovered pro-oncogene in the Wnt/β-catenin signaling pathway, and its abnormal expression is a new mechanism of tumor development^14^. BCL9 was found to be abnormally expressed in a variety of human tumor tissues, such as multiple myeloma^26^, colorectal cancer^27^, hepatocellular carcinoma^28^, and breast cancer^29^. Whether there are gene mutations in the Wnt/β-catenin pathway or not, BCL9 can enhance the gene transcriptional activity mediated by β-catenin, thereby promoting tumor proliferation, migration and invasion^26^. Our study found that, miR-30a-5p binds to the 3′UTR region of the BCL9 gene and inhibits its translation, thereby affecting the activity of nuclear TCF/LEF transcription factor and transcription of β-catenin downstream target genes, thus ultimately affecting the growth and migration functions of *H. pylori*-infected gastric cancer cells.

It is well known that the miR-30a precursor is presented *in vivo* in the form of complementary stem-loop structure including miR-30a-3p and miR-30a-5p, which can be processed to generate two separate miRNAs and play their respective biological function. miR-30a is located on human chromosome 6q13, and is an important member of the miR-30 family. Many reports have shown that, miR-30a had low expression levels in breast cancer^30^, bone tumors^31^, non-small cell lung cancer^32^, liver cancer^33^ and other malignant tumors. miR-30a can regulate the proliferation, apoptosis, invasion, migration and other biological function of different tumor cells^34^. In our study, we found that the double-stranded miR-30a precursor could be processed to generate single-stranded miR-30a-3p and miR-30a-5p, and showed enhanced inhibitory effect on the expression of COX-2 and BCL9 compared to the individual effect of miR-30a-3p or miR-30a-5p, as well as the regulating effect on LEF/TCF promoter activity and transcription of downstream target genes such as Cyclin D1, MMP7, CD44, and c-Myc. In addition, the miR-30a precursor showed an increased inhibitory effect on the growth and migartion of *H. pylori*-infected gastric cancer cells.

Since miR-30a has very important regulatory effect on the growth and migration of *H. pylori*-infected gastric cancer cells, we constructed the miR-30a knockout mouse model to further investigate the function of miR-30a *in vivo*. In recent years, many genome-editing technologies have emerged, including zinc-finger nucleases (ZFNs), transcription activator-like effector nucleases (TALENs) and the sequence-specific CRISPR/Cas9 nuclease system^35^. CRISPR/Cas9 technology, as an exciting new genetic perturbation system that enables the targeted modification of the DNA sequence itself, overcomes the shortcomings of existing technology, including long period, heavy workload, high difficulty, and low efficiency in the construction of gene knockout mice^36, 37^. Using CRISPR/Cas9 technology, we successfully achieved the production of miR-30a knockout homozygous mice. In view of this, we further investigated the effect of *H. pylori* infection on miR-30a knockout mice. Not surprisingly, miR-30a knockout did not affect the growth and development of the mice, and had little effect on the *H. pylori* colonization rates of mice. However, *H. pylori*-infected miR-30a knockout mice showed increased incidence of chronic gastritis, chronic atrophic gastritis, atypical hyperplasia, and other precancerous lesions and adenocarcinoma manifestations in the antral or gastric mucosa of mice. Although there are several reports stating the very low frequency of gastric cancer progression in C57BL/6 mice^38–40^, our results demonstrated that the percentage of adenocarcinoma was 5% (1/20) to 15% (3/20), and miR-30a KO increased the percentage of adenocarcinoma to more than 20% (4/20). Different strains of *H. pylori* for infecting C57BL/6 mice (*H. pylori* S11 for Kim *et al*. and *H. pylori* NCTC11637 for us) might be the first cause of different percentage of adenocarcinoma from previous models. Moreover, our method for infecting C57BL/6 mice was different from that described by Kin *et al*. For *H. pylori* S11, the mice were dosed three times for a 3-day period with 100 μL of 1 × 10^7^ CFU/mL bacterial suspension (approximately 1 × 10^6^ CFU) by oro-gastric tube. For *H. pylori* NCTC11637, each mouse was fed with 1 mL *H. pylori* (1 × 10^9^ CFU/mL) every other day, for a total of 5 times. All the experimental groups of mice were pretreated with no food for 12 h and no drinking for 4 h. After gavage administration, the mice were kept with no food and drinking for another 4 h.

Moreover, *H. pylori*-infected miR-30a knockout mice showed up-regulated expression of above mentioned COX-2 and BCL9, as well as the growth and metastasis associated genes expression such as VEGF and CD34 (marker of microvessel density, MVD). In addition, the IHC staining and real-time PCR results of COX-2, BCL9, VEGF, CD34, miR-30a-5p and miR-30a-3p in human tissue samples from patients with *H. pylori* gastritis, *H. pylori*-related gastric cancer and healthy controls were also in agreement with those findings *in vitro* and *in vivo*. The expression of COX-2 was closely associated with the expression of the VEGF and CD34 genes. Uefuji *et al*.^41^ found that, 42 cases of gastric cancer patients with positive COX-2 expression showed significantly higher CD34 expression than those with negative COX-2 expression, implying that over-expression of COX-2 was highly associated with tumor angiogenesis. Yu *et al*.^42^ showed in gastric cancer that, COX-2 expression and VEGF expression were significantly correlated, and gastric cancer patients with positive expression of COX-2 and VEGF also exhibited significantly higher MVD than those patients with negative expression. Tang *et al*.^43^ and Lazăr *et al*.^44^ also found in gastric cancer that COX-2 expression was positively correlated with VEGF expression. Taken together, our results indicated that, the function of miR-30a in the development of *H. pylori*-induced gastric cancer was associated closely with the regulation of COX-2, BCL9, VEGF and CD34.

miR-30a is a newly found small molecular that affects the *H. pylori*-induced gastric cancer, and its lower expression in *H. pylori* gastritis and gastric cancer showed its potential as a clinical diagnostic. However, our current study does not provide the mechanism by which *H. pylori* downregulates miR-30a (miR-30a-3p and miR-30a-5p), which is required to be addressed in future investigation. Since the dysregulation of oncogenes or suppressors such as miR-30a and the abnormal activation of oncogenic pathways such as Wnt/β-catenin have been achieved before *H. pylori* eradication, to seek more effective drugs targeting the involved genes or signaling pathway is more real in therapy for *H. pylori*-related gastric cancer.

In summary, the results of our *in vitro* and *in vivo* experiments indicate that, miR-30a, the complementary sequence of miR-30a-3p and miR-30a-5p, functions as a tumor suppressor by double-targeting COX-2 and BCL9 in *H. pylor*i-infected gastric cancer cells and significantly affects the development of *H. pylori*-induced gastric cancer, shedding new light on the mechanisms underlying *H. pylori*-associated gastric cancer (Fig. S8).

## Methods

### Ethics Statement

All experimental procedures were performed in strict accordance with the guidelines for the care and use of animals of Shanghai University of Traditional Chinese Medicine. All animal experimental procedures were approved by the Animal Care and Use Committee of Shanghai University of Traditional Chinese Medicine (No. 20140516). Every effort was made to minimize animal pain, suffering, and distress. The human specimens in this study were sanctioned by the local ethics committee at Shuguang Hospital, Shanghai University of Traditional Chinese Medicine (No. 2014-382-26).

### Human tissues

Tumor tissues were isolated from 60 gastric cancer patients (41 males and 19 females with an average age of 54 years old, with *H. pylori* infection history or *H. pylori* detected in surgical specimens, between stage II and stage IV) who underwent tumor resection at Shuguang Hospital, Shanghai University of Traditional Chinese Medicine, between 2010 and 2015, and all tissues were examined histologically. None of the patients received preoperative treatment, including chemotherapy or radiotherapy. The biopsies of chronic gastritis (60 cases) and healthy controls (60 cases) were obtained from outpatients during endoscopic procedure at Shuguang Hospital, Shanghai University of Traditional Chinese Medicine. Informed consent was obtained from all patients enrolled in this study. All methods involving experiments on humans and/or the use of human tissue samples were performed in accordance with the relevant guidelines and regulations.

### Cell and *H. pylori* culture

Human MKN45 and SGC-7901 gastric cancer cell lines was purchased from the American Type Culture Collection (ATCC, Manassas, VA, USA), and cultured in RPMI 1640 medium containing 10% fetal bovine serum, 100 U/mL penicillin and 100 μg/mL streptomycin, at 37 °C, 5% of CO_2_, and saturated humidity. HEK293T cell line was purchased from ATCC and cultured in Dulbecco’s Modified Eagle Medium (DMEM). International standard strain *H. pylori* NCTC11637 (containing cacA and cagA gene) was purchased from the Institute of Digestive Diseases, Shanghai Renji Hospital (Shanghai, China).

### Cell transfection

Cell transfection was performed using the HilyMax kit (DOJINDO, Japan) according to the manufacturer’s instructions. The double-stranded miRNA mimics and their respective negative control miRNA mimic (GeneChem, Shanghai, China) were introduced into cells at a final concentration of 50 nM (Table S1).

### *H. pylori* infection

*H. pylori* was cultured on Columbia agar plates (Oxoid, Basingstoke Hampshire, UK) containing 5% sheep blood, at 37 °C under microaerophilic conditions. Colonies were identified as *H. pylori* by morphology, Gram staining, positive reactions to oxidase, catalase, and urease activities. *H. pylori* were suspended in phosphate buffered saline (PBS) to estimate the concentration by spectrophotometry (OD600 nm, A600 = 1 × 10^8^ CFU/mL). According to the concentration proportion of *H. pylori*:cell = 100:1 (antibiotic-free medium with 10% fetal bovine serum was used to dilute *H. pylori*), and the *H. pylori* solutions were added to infect MKN45 or SGC-7901 cells.

### Cell proliferation assay

MTT was applied to test cell viability by measuring the absorbance at 490 nm, and all the MKN45 cells were pretreated with *H. pylori* for 12 h before the treatment with different miRNA mimics or inhibitors. All assays were performed in triplicate and independently repeated three times.

### miRNA microarray and bioinformatics analysis

Trizol was used to extract total RNAs from MKN45 or *H. pylori* MKN45 cells. The concentrations, purities and integrities of RNAs were determined using Nanodrop spectrophotometer and denaturing agarose gel electrophoresis with formaldehyde. To acquire the fluorescent probe for hybridization, the miRNAs were labeled with Hy3™ or Hy5™ fluorophore using miRCURY™ Array Power labeling kit. Under the standard hybridization conditions, the former labeled probe hybridized with miRCURY™ microarray. GenePix 4000B microarray scanner was used to scan the fluorescence intensity of the microarray, and the data was applied for further bioinformatics analysis.

### Quantitative RT-PCR

Total RNAs and miRNAs were extracted using Trizol and miRNAs Extract Kit (TaKaRa, Dalian, China), respectively, according to manufacturer’s instructions. Reverse transcription was performed using PrimeScriptTM RT-PCR Kit (TaKaRa, Dalian, China). PCR primers were listed in Table S2. Real-time PCR was performed in the ABI 7300 System using Premix Ex Taq (TaKaRa, Dalian, China). GAPDH and U6 were used as internal references.

### Dual luciferase activity detection

Approximately 8,000 HEK293T cells per well or 12,000 MKN45 cells per well were plated into 96-well plates and were cotransfected with 50 nmol/L of miRNA mimic (or NC), 50 ng luciferase reporter (pmirGLO-COX-2-3′UTR, or pmirGLO-BCL9-3′UTR), or 50 ng luciferase reporter (pmirGLO-COX-2-3′UTR-mut, or pmirGLO-BCL9-3′UTR-mut) using HilyMax kit (DOJINDO, Japan). Previously constructed plasmid pGL3-basic-LEF/TCF promoter and plasmid pRL-SV40 were used as the irrelevant and positive controls, respectively^45^. After 48-hour incubation, according to the instructions of dual luciferase activity detection kit (Promega, USA), the fluorescence intensity of COX-2 3′UTR or BCL9-3′UTR was measured.

### Western blot

Cells were washed with PBS for three times, added with cell lysis solution, and incubated in the ice bath for 15 minutes. The total protein was obtained from the cell lysate by centrifugation. Protein samples were loaded and separated by SDS-PAGE electrophoresis, transferred to PVDF membranes, and blocked in 5% skimmed milk prior to incubation with the primary and secondary antibodies. After washing with PBST (PBS containing 0.2% Tween-20) for three times, the resulting immunocomplexes were visualized by enhanced ECL chemiluminescence, followed by photographing and quantitative analysis.

### Transwell analysis

A total of 2.5 × 10^5^ MKN45 cells (in 100 µL RPMI 1640 with 0.5% FCS) or MKN45 cells pretreated with *H. pylori* for 12 h were seeded into the upper part of the transwell chamber (Corning, Corning, NY, USA). For migration analysis, in the lower part of the transwell chamber, 600 µL RPMI 1640 with 15% FCS and 10 µg/mL fibronectin was added. 24 hours later, the migrated cells were analyzed by crystal violet staining and photographed with the DMI3000B inverted microscope (Leica, Germany). For invasion analysis, 100 µL matrigel (BD, Biosciences, San Jose, CA, USA) was pre-laid into the bottom of the transwell chamber before MKN45 cells were seeded, and the subsequent procedures were similar as for the migration analysis. The exception for invasion analysis is the co-culture time of 48 hours, but not 24 hours.

### Soft agar colony formation assay

MKN45 cells or MKN45 cells pretreated with *H. pylori* for 12 h were plated in 24-well culture plates that contained two layers of soft agar (Corning, USA) at a density of 500 cells/well and allowed to grow undisturbed for 14 days, at 37 °C, high humidity, and 5% CO_2_. The top and bottom layers were 0.33% and 0.8% low melt agarose (Bio-Rad, Hercules, CA, USA) in 5% RPMI 1640 medium, respectively. Colonies were counted by two investigators (QJ and XL).

### Cell immunofluorescence

Cells were fixed with methanol, blocked with 5% BSA, and perforated with 0.1% TRITON X-100. Next, the cells were stained with primary antibody followed by Cy3-conjugated goat anti-rabbit IgG (Millipore, Germany). Nuclear staining was performed with DAPI. Cells were imaged with a TCS SP2 spectral confocal system (Leica, Germany).

### Construction of miR-30a knockout mouse model using CRISPR/Cas9 technology

To knockout the miR-30a gene *in vivo*, two primers were designed and synthesized, wherein the first primer comprised the T7 promoter sequence for *in vitro* transcription, the miR-30a target-specific sequence, and a partial sequence for the gRNA (guilding RNA) backbone. The other primer contained the remaining sequences of the gRNA backbone. There were the paired sequences between the 3′ end of the above two primers. By PCR amplification, the *in vitro* transcription template DNA was obtained. In addition, the two primers with the 5′phosphate groups were synthesized as follows: NcoI-SP6-Sense: 5′-CATGGATTTAGGTGACACTATAGAAGAGC-3′, NcoI-SP6-Antisense: 5′-CATGGCTCTTCTATAGTGTCACCTAAATC-3′. After mixing in equal proportions, above two primers were annealed to form the double-stranded DNA, each end with NcoI sticky end and the middle SP6 promoter sequence. At the same time, the pX260 plasmid was enzyme digested with NcoI, dephosphorylated, and then connected with above annealed products. After transformation and DNA sequencing, the Cas9 plasmid for *in vitro* SP6 transcription can be obtained.

gRNA template amplified by PCR was purified by phenol-chloroform extraction and ethanol precipitation, following by transcription using *in vitro* T7 transcription kit and recovery by phenol-chloroform extraction and isopropanol precipitation. Above constructed plasmid pX260 (contains only a NotI restriction site, and locates behind the Cas9 downstream polyA sequence) was digested by NotI, transcribed *in vitro* with MACHINE® SP6 *in vitro* transcription kit, and purified by lithium chloride precipitation.

Zygotes for microinjection were originated from superovulated female C57BL/6 mouse after mating with male C57BL/6 mouse. Prior to microinjection, the zygotes were cultured in KSOM embryo medium. TE buffer was used to mix gRNA and Cas9 mRNA, and dilute to the appropriate concentration. Above solution was injected into the cytoplasm of single cell zygote with microinjection needle by Eppendorf transferMan NK2 micromanipulation device. Immediately after microinjection, the zygotes were transplanted into pseudopregnant female mice. The female mice were housed in the SPF environment with 12 hours lighting rhythm, enough food and drinking.

After one week, the toes of the newborn mice were cut and digested overnight at 55 °C, and the genomic DNAs were extracted using phenol-chloroform extraction method. PCR were performed to identify the success ratio and efficiency of miR-30a knockout. PCR primers were listed in Table S3. PCR reaction conditions: 94 °C, 3 min; 94 °C, 30 s; 56 °C, 30 s; 72 °C, 45 s; 72 °C, 8 min, 30 cycles. 5 μl PCR products were drawn for agarose gel (1.5%) electrophoresis to characterize the targeting band. Next, the PCR product was ligated into T vector, and at least 6 clones of each mouse were picked and sequenced. The sequencing results were compared with the wild-type miR-30a gene. According to the compared results, F0 generation mice with miR-30a knockout efficiently were screened out. The above identified F0 generation mice were mated with wild type mice to obtain F1 generation mice. The heterozygous mice were obtained from the posterity of F1 generated mice by PCR amplification and genotyping identification. The homozygous F2 generation mice with miR-30a knockout were obtained by two-two mating of above heterozygous mice.

### Production of *H. pylori* infected miR-30a knockout mouse model

The *H. pylori* colonies were scraped and adjusted the concentration to 1 × 10^9^ CFU/ml. Each mouse was fed with 1 ml* H. pylori* (1 × 10^9^ CFU) every other day, and in total for 5 times. All the experimental groups of mice were pre-treatment with no food for 12 hours and no drinking for 4 hours. After gavage administration, the mice were kept with no food and drinking for another 4 hours. After 72 weeks of feeding *H. pylori*, all mice were sacrificed. Opened laparotomy in the middle, took out the whole stomach (including the antrum, gastric body, duodenum, etc.), cut along the greater curvature, and exposed the gastric mucosa. Then, firstly, visually observed the general characterization of mice gastric mucosa, and isolated the gastric mucosa. One third of the gastric mucosa was used to do rapid urease test, another one third was fixed in 10% formalin for histopathological examination and Giemsa staining, and the last one third was preserved in DEPC H_2_O for RNA and protein extraction.

### HE staining and IHC staining

Above paraffin-embedded tissues were sectioned for HE (hematoxylin and eosin) staining and IHC (immunohistochemistry) staining. For IHC analysis, the experiments were performed using the first antibody, HRP-conjugated secondary antibody, and DAB (diaminobenzidine) detection reagents. The DMI3000B microscope connected to the digital imaging system was applied for photograph and further analysis. All the results were evaluated and classified blindly by two investigators (LLW and ZYW) from pathology department of Shuguang Hospital, Shanghai University of Traditional Chinese Medicine. The staining was scored as follows: the distribution of the signal (distribution score) was scored as 0 (0–5%), 1 (6–25%), 2 (26–50%), 3 (51–75%), or 4 (76–100%) to indicate the percentage of positive cells in one tissue. The intensity of the signal (intensity score) was scored as 0 (no signal), 1 (weak), 2 (moderate), or 3 (marked). The distribution score and intensity score were then multiplied to attain a total score.

### Statistical analysis

All the data were presented as mean ± standard deviation and analyzed with SPSS20.0 software. Statistic comparison was performed using the Student’s t-test or Mann-Whitney U test, as appropriate. *p* < 0.05 was considered statistically significant, and *p* < 0.01 was considered as statistically highly significant.

## Electronic supplementary material

Supplementary Information
