# Supplementary material for: miR-30a acts as a tumor suppressor by double-targeting COX-2 and BCL9 in H. pylori gastric cancer models
Source: Sci Rep. 2017 Aug 2;7:7113. doi: 10.1038/s41598-017-07193-w (PMC5540978; doi:10.1038/s41598-017-07193-w)
Supplement: Supplementary file 1 — Supplementary Information [file 41598_2017_7193_MOESM1_ESM.pdf]

miR-30a acts as a tumor suppressor by double-targeting COX-2 and BCL9 in *H. pylori* gastric cancer models

Xuan Liu[1,]*, Qing Ji[1,]*, Chengcheng Zhang[2], Xiaowei Liu[3], Yanna Liu[4], Ningning Liu[1], Hua Sui[1], Lihong Zhou[1], Songpo Wang[5], Qi Li[1]

Supplementary tables

Table S1 Mimic and inhibitor sequences for different miRNAs

Gene	Sequences
miR-29b-1* mimics	5-GCUGGUUUCAUAUGGUGGUUUAGA-3
miR-3125 mimics	5-UAGAGGAAGCUGUGGAGAGA-3
miR-30a-3p mimics	5-CUUUCAGUCGGAUGUUUGCAGC-3
miR-30a-5p mimics	5-UGUAAACAUCCUCGACUGGAAG-3
miR-340 mimics	5-UUAUAAAGCAAUGAGACUGAUU-3
miR-301a mimics	5-CAGUGCAAUAGUAUUGUCAAAGC-3
miR-451 mimics	5-AAACCGUUACCAUUACUGAGUU-3
miR-67 mimics	5-UCACAACCUCCUAGAAAGAGUAGA-3
Anti-miR-29b-1*	5-UCUAAACCACCAUAUGAAACCAGC-3
Anti- miR-3125	5-UCUCUCCACAGCUUCCUCUA-3
Anti- miR-30a-3p	5-GCUGCAAACAUCCGACUGAAAG-3
Anti- miR-30a-5p	5-CUUCCAGUCGAGGAUGUUUACA-3
Anti- miR-340	5-AAUCAGUCUCAUUGCUUUAUAA-3
Anti- miR-301a	5-GCUUUGACAAUACUAUUGCACUG-3
Anti- miR-451	5-AACUCAGUAAUGGUAACGGUUU-3
Scramble	5-AAGGCAAGCUGACCCUGAAGU-3

Table S2 Primer sequences for qRT-PCR

Gene	Primer sequences
MALAT1	F: 5-GCTCTGTGGTGTGGGATTGA-3
	R: 5-GTGGCAAAATGGCGGACTTT-3
COX-2	F: 5-GGCCATGGGGTGGACTTAAA-3
	R: 5-CCCCACAGCAAACCGTAGAT-3
BCL9	F: 5-TCAGCCCAATGGGAATGACC-3
	R: 5-GGCCATTGTGAGGGAATGGA-3
GAPDH	F: 5-GGTGGTCTCCTCTGACTTCAACA-3
	R: 5-CCAAATTCGTTGTCATACCAGGAAATG-3
miR-29b-1*	F: 5-ACACTCCAGCTGGG GCTGGTTTCATATGG-3
	R: 5-CTCAACTGGTGTCGTGGAGTCGGCAATTCAGTT GAGTCTAAACCTCC-3
miR-3125	F: 5-ACACTCCAGCTGGGTAGAGGAAGCTGTGG-3

	R: 5-CTCAACTGGTGTCGTGGAGTCGGCAATTCAGTT GAGTCTCTCCACAGC-3
miR-30a-3p	F: 5-ACACTCCAGCTGGGCTTTCAGTCGGATG-3
	R: 5-CTCAACTGGTGTCGTGGAGTCGGCAATTCAGTT GAGGCTGCAAACATC-3
miR-30a-5p	F: 5-ACACTCCAGCTGGGTGTAAACATCCTCGAC-3
	R: 5-CTCAACTGGTGTCGTGGAGTCGGCAATTCAGTT GAGCTTCCAGTCGAGG-3
miR-340	F: 5-ACACTCCAGCTGGGTTATAAAGCAATGAGAC-3
	R: 5-CTCAACTGGTGTCGTGGAGTCGGCAATTCAGTT GAGAATCAGTCTCATTGC-3
miR-301a	F: 5-ACACTCCAGCTGGGCAGTGCAATAGTATTG-3
	R: 5-CTCAACTGGTGTCGTGGAGTCGGCAATTCAGTT GAGGCTTTGACAATACTA-3
miR-451	F: 5-ACACTCCAGCTGGGAAACCGTTACCATTAC-3
	R: 5-CTCAACTGGTGTCGTGGAGTCGGCAATTCAGTT GAGAACTCAGTAATGG-3
U6	F: 5-CTCGCTTCGGCAGCACA-3
	R: 5-AACGCTTCACGAATTTGCGT-3

Table S3 Primer sequences for PCR identification of miR-30a in mice

**Primer sequences**
Sense primer: 5-CTTTTCTGCTTTCCTCCTGAT-3
Anti-sense primer: 5-GTTTAATTTATCTTTTCCACCCA-3

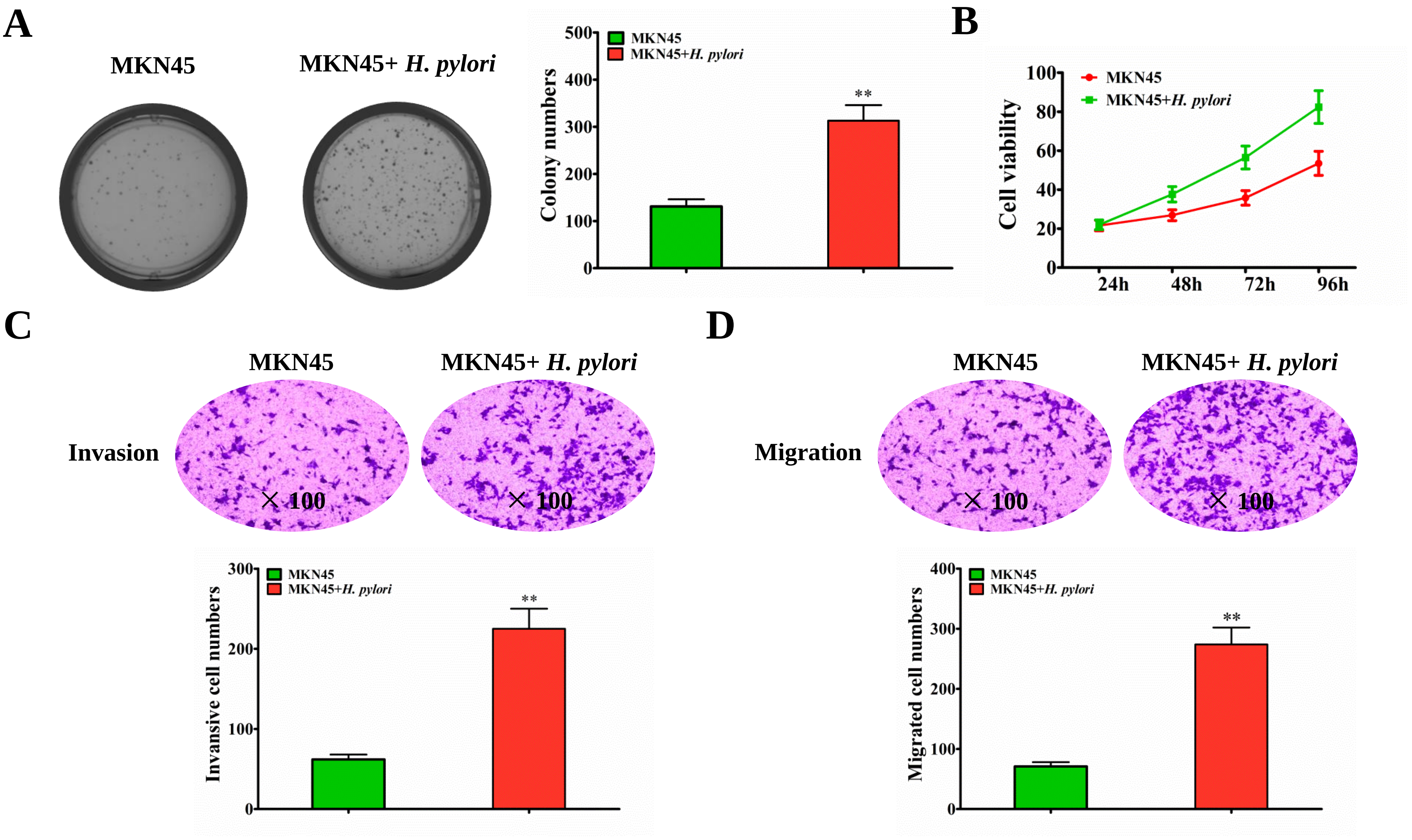
A
MKN45
MKN45+ H. pylori
Colony numbers
MKN45
MKN45+H. pylori
**
B
Cell viability
MKN45
MKN45+H. pylori
24h
48h
72h
96h
C
MKN45
MKN45+ H. pylori
Invasion
× 100
Invansive cell numbers
D
MKN45
MKN45+ H. pylori
Migration
× 100
Migrated cell numbers

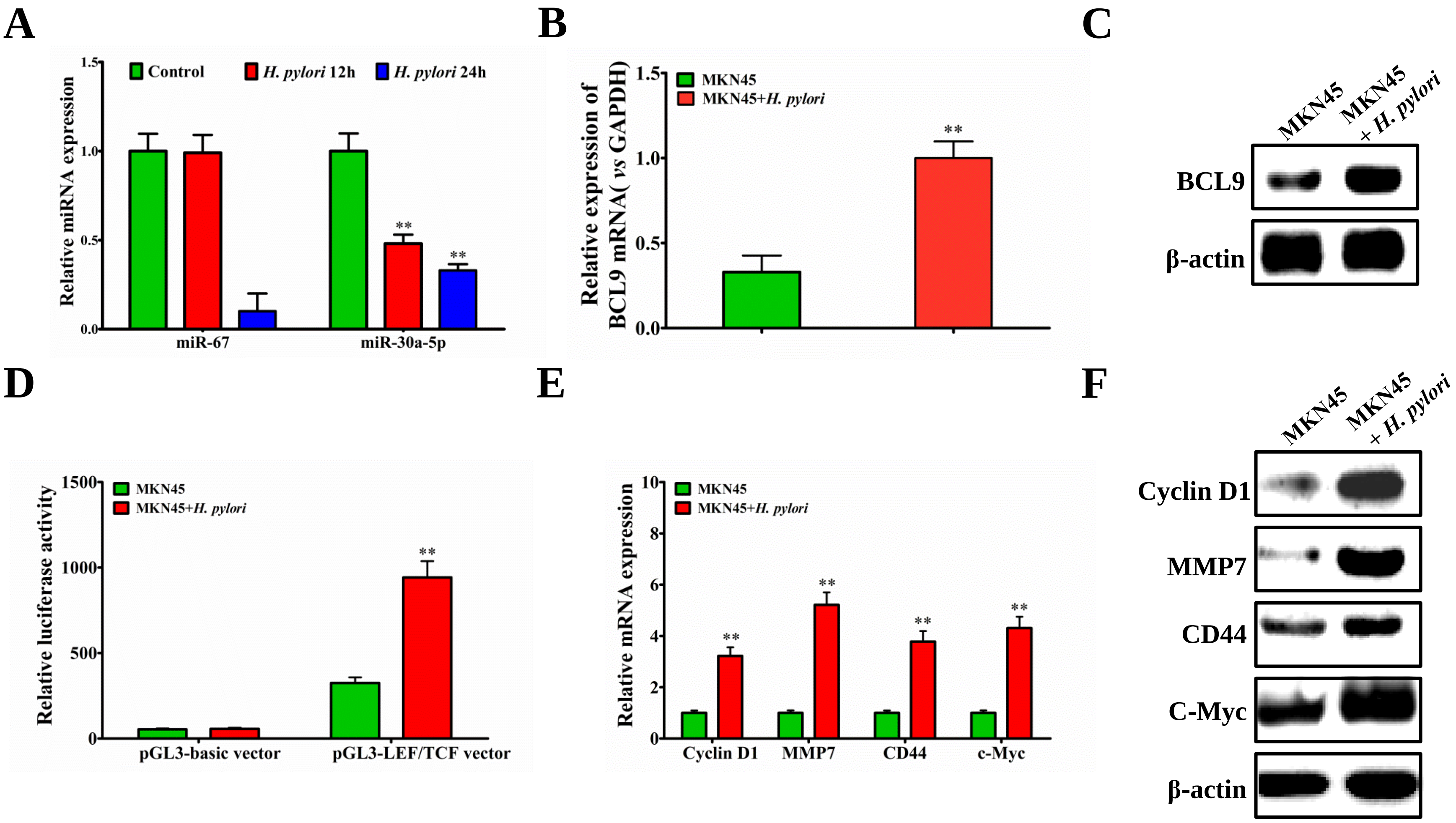
A
Control
H. pylori 12h
H. pylori 24h
Relative miRNA expression
1.5
1.0
0.5
0.0
**
**
miR-67
miR-30a-5p
B
MKN45
MKN45+H. pylori
Relative expression of BCL9 mRNA(vs GAPDH)
1.5
1.0
0.5
0.0
**
C
MKN45
MKN45 + H. pylori
BCL9
β-actin
D
MKN45
MKN45+H. pylori
Relative luciferase activity
1500
1000
500
0
**
pGL3-basic vector
pGL3-LEF/TCF vector
E
MKN45
MKN45+H. pylori
Relative mRNA expression
10
8
6
4
2
0
**
**
**
**
Cyclin D1
MMP7
CD44
c-Myc
F
MKN45
MKN45 + H. pylori
Cyclin D1
MMP7
CD44
C-Myc
β-actin

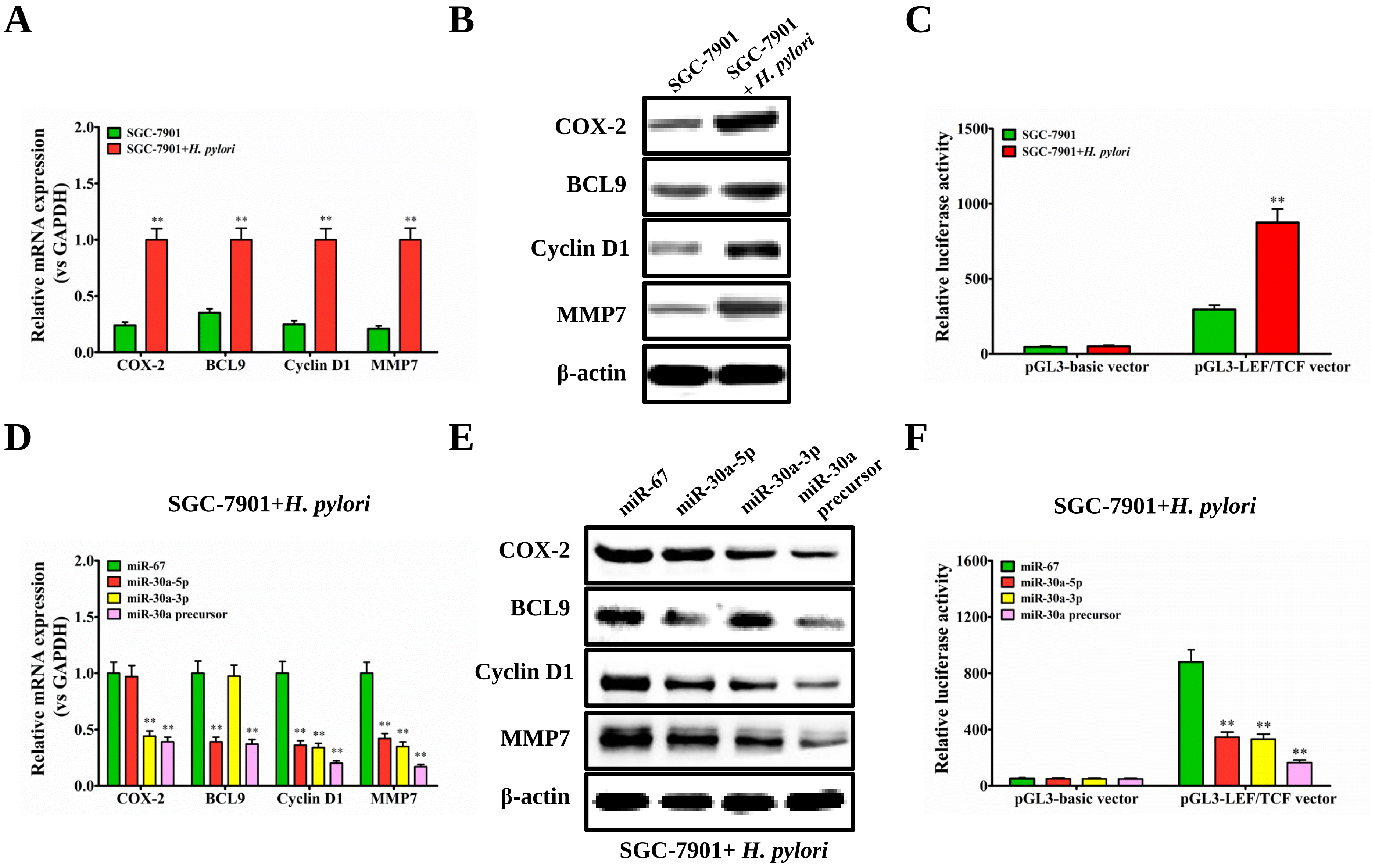
A
Relative mRNA expression (vs GAPDH)
SGC-7901
SGC-7901+H. pylori
2.0
1.5
1.0
0.5
0.0
**
COX-2
BCL9
Cyclin D1
MMP7
B
SGC-7901
SGC-7901 + H. pylori
COX-2
BCL9
Cyclin D1
MMP7
β-actin
C
Relative luciferase activity
SGC-7901
SGC-7901+H. pylori
1500
1000
500
0
**
pGL3-basic vector
pGL3-LEF/TCF vector
D
SGC-7901+H. pylori
Relative mRNA expression (vs GAPDH)
miR-67
miR-30a-5p
miR-30a-3p
miR-30a precursor
2.0
1.5
1.0
0.5
0.0
COX-2
BCL9
Cyclin D1
MMP7
E
miR-67
miR-30a-5p
miR-30a-3p
miR-30a precursor
COX-2
BCL9
Cyclin D1
MMP7
β-actin
SGC-7901+ H. pylori
F
SGC-7901+H. pylori
Relative luciferase activity
miR-67
miR-30a-5p
miR-30a-3p
miR-30a precursor
1600
1200
800
400
0
pGL3-basic vector
pGL3-LEF/TCF vector

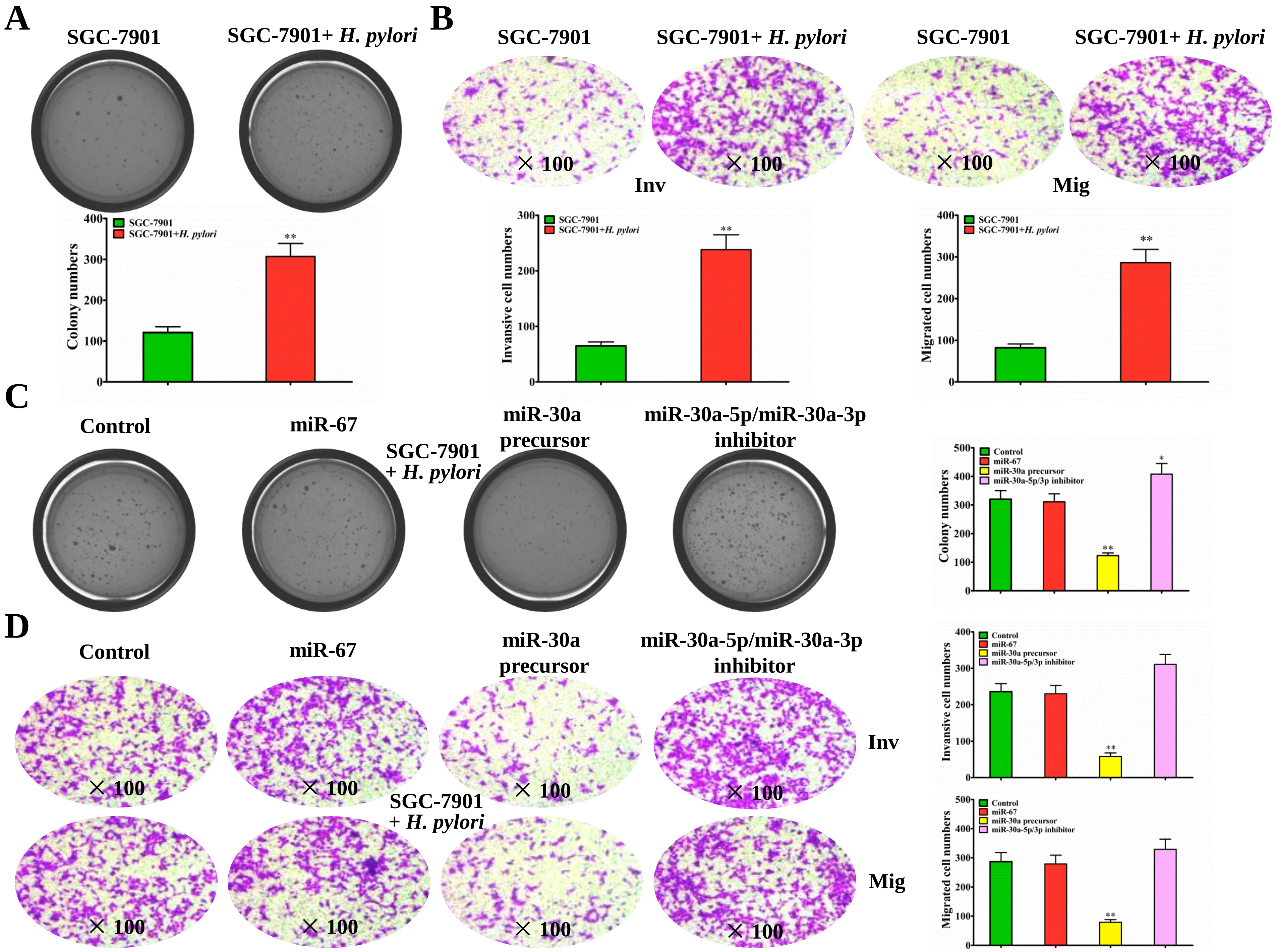
A
SGC-7901
SGC-7901+ H. pylori
Colony numbers
SGC-7901
SGC-7901+H. pylori
**
B
SGC-7901
SGC-7901+ H. pylori
SGC-7901
SGC-7901+ H. pylori
× 100
Inv
Mig
Invansive cell numbers
Migrated cell numbers
C
Control
miR-67
SGC-7901
+ H. pylori
miR-30a
precursor
miR-30a-5p/miR-30a-3p
inhibitor
Control
miR-67
miR-30a precursor
miR-30a-5p/3p inhibitor
D
Control
miR-67
miR-30a
precursor
miR-30a-5p/miR-30a-3p
inhibitor
SGC-7901
+ H. pylori
Inv
Mig

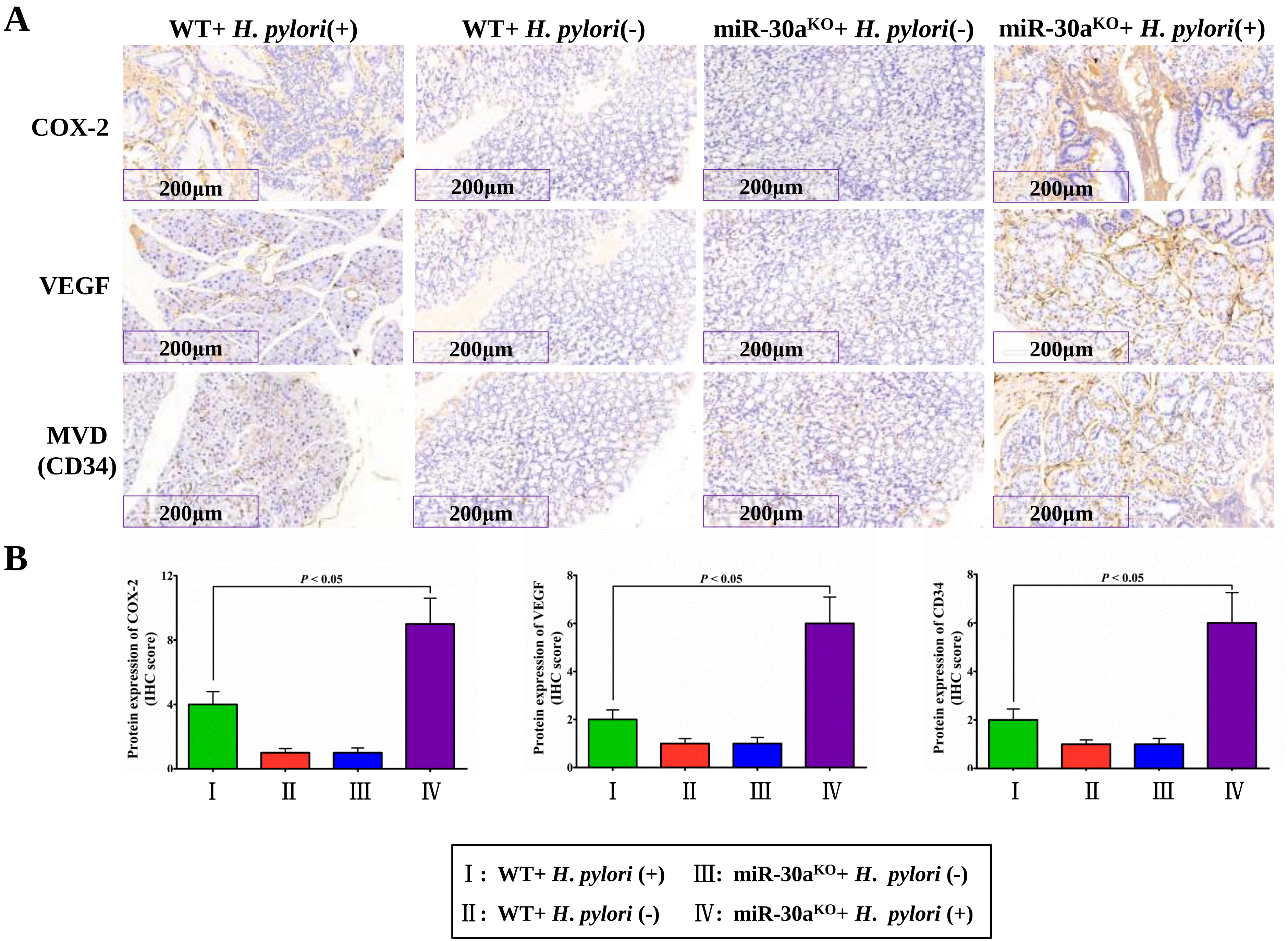
A
WT+ H. pylori(+)
WT+ H. pylori(-)
miR-30aKO+ H. pylori(-)
miR-30aKO+ H. pylori(+)
COX-2
VEGF
MVD (CD34)
200μm
B
Protein expression of COX-2 (IHC score)
Protein expression of VEGF (IHC score)
Protein expression of CD34 (IHC score)
P < 0.05
Ⅰ Ⅱ Ⅲ Ⅳ
Ⅰ: WT+ H. pylori (+)
Ⅱ: WT+ H. pylori (-)
Ⅲ: miR-30aKO+ H. pylori (-)
Ⅳ: miR-30aKO+ H. pylori (+)

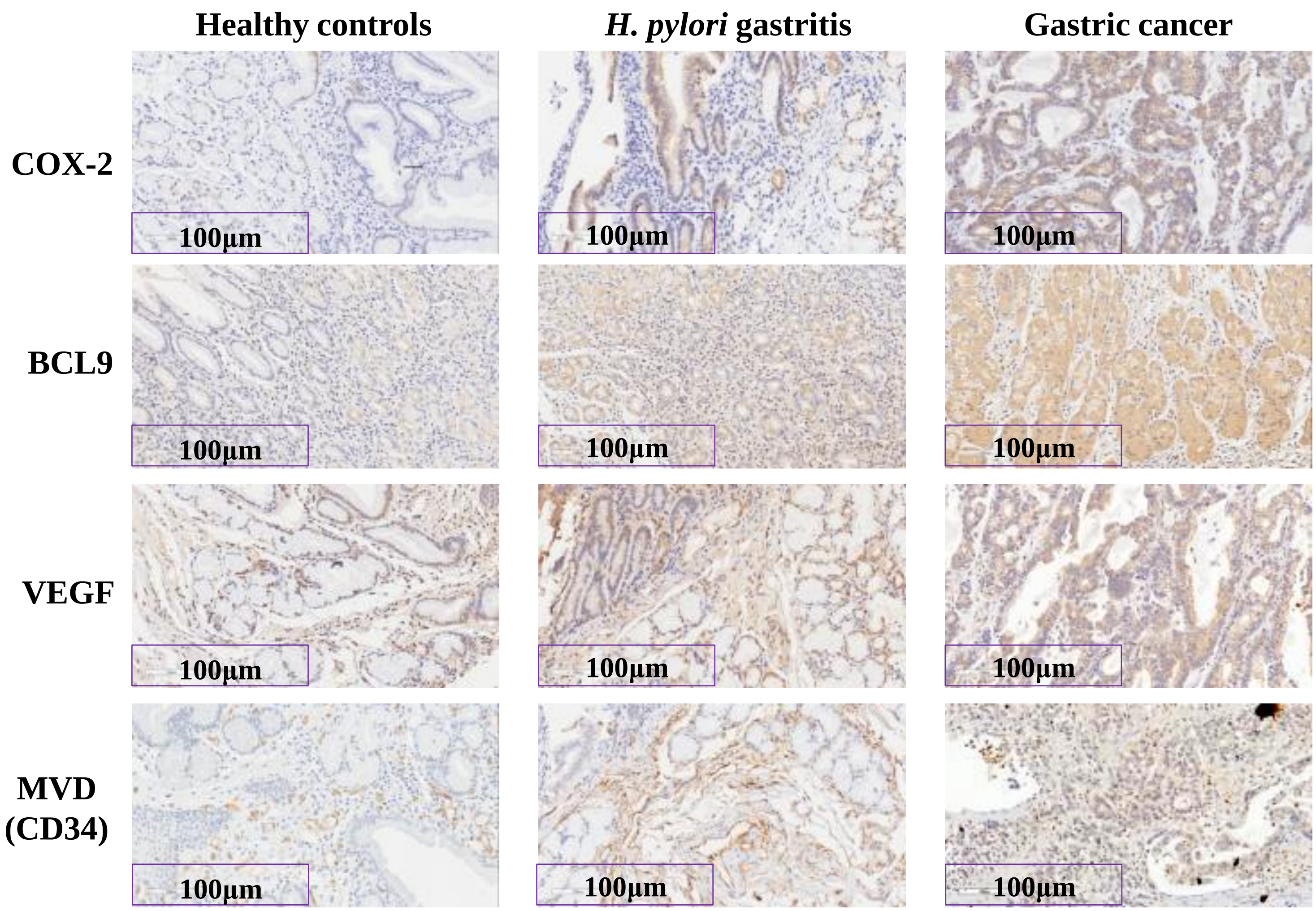

Healthy controls
H. pylori gastritis
Gastric cancer
COX-2
BCL9
VEGF
MVD (CD34)
100µm

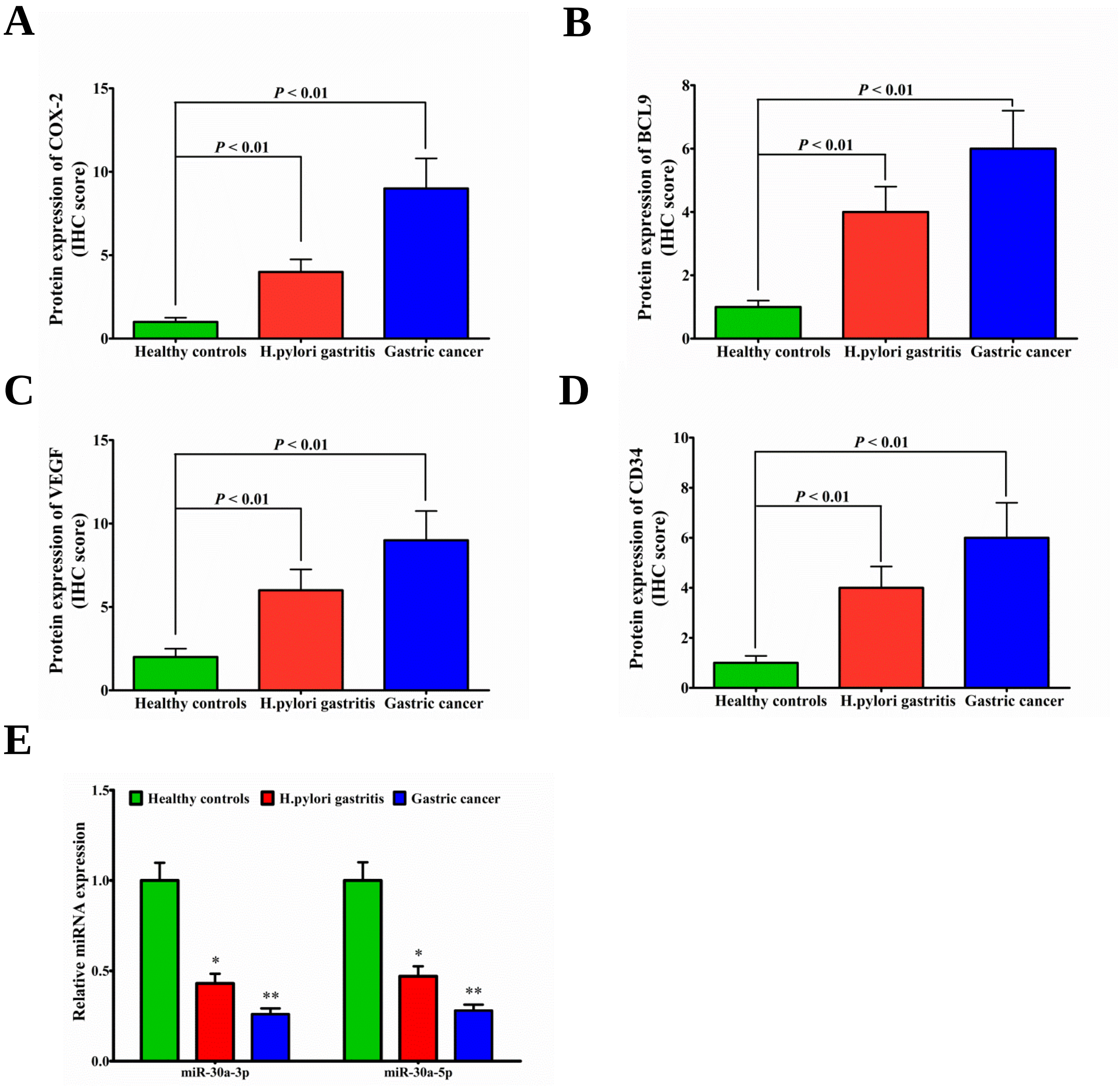
A
Protein expression of COX-2 (IHC score)
P < 0.01
P < 0.01
Healthy controls
H.pylori gastritis
Gastric cancer
B
Protein expression of BCL9 (IHC score)
P < 0.01
P < 0.01
Healthy controls
H.pylori gastritis
Gastric cancer
C
Protein expression of VEGF (IHC score)
P < 0.01
P < 0.01
Healthy controls
H.pylori gastritis
Gastric cancer
D
Protein expression of CD34 (IHC score)
P < 0.01
P < 0.01
Healthy controls
H.pylori gastritis
Gastric cancer
E
Relative miRNA expression
Healthy controls
H.pylori gastritis
Gastric cancer
miR-30a-3p
miR-30a-5p

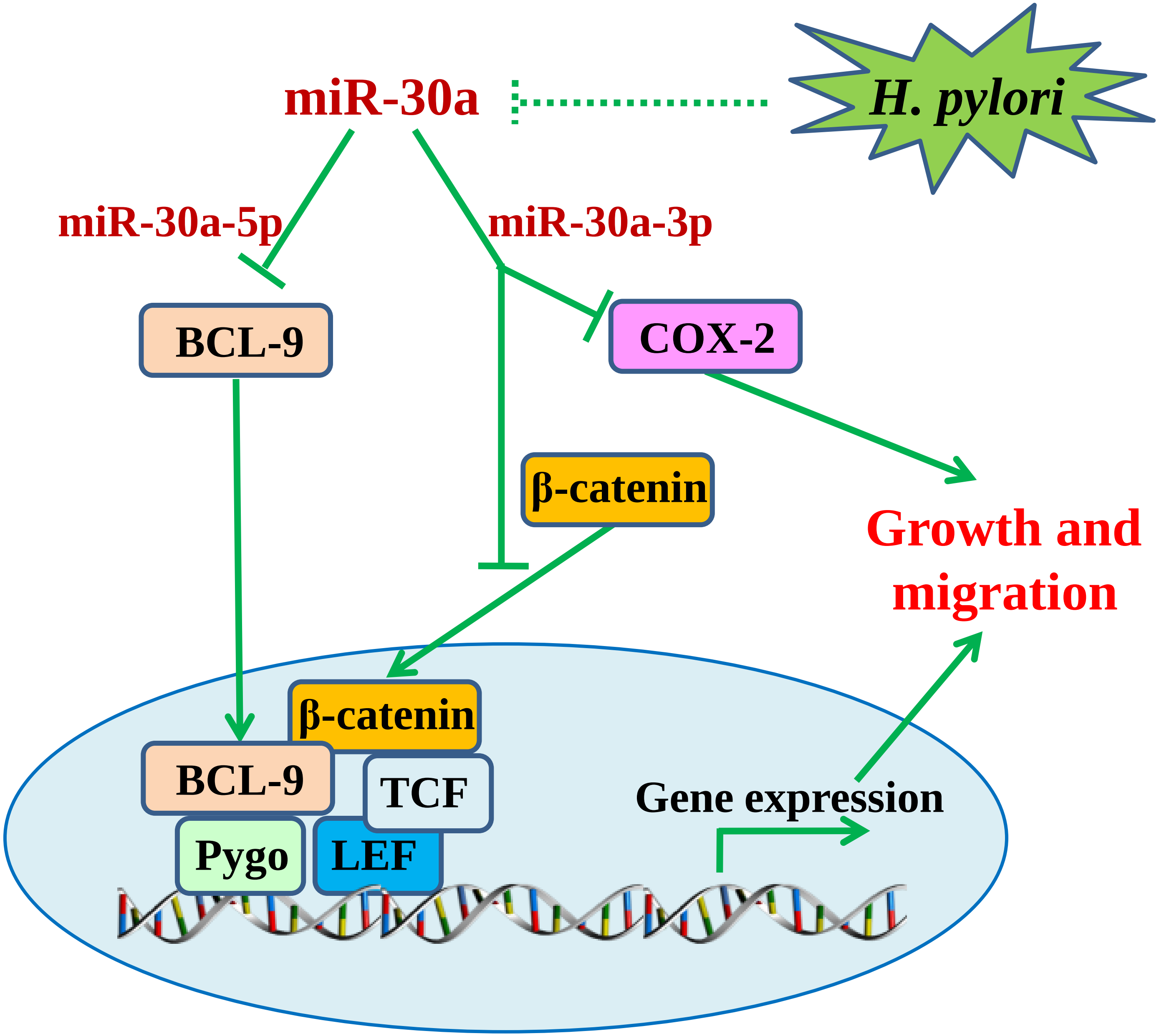
miR-30a
H. pylori
miR-30a-5p
miR-30a-3p
BCL-9
COX-2
β-catenin
Growth and
migration
β-catenin
BCL-9
TCF
Gene expression
Pygo
LEF

Supplementary figure legends

Figure S1 | Effect of *H. pylori* on the growth and migration of gastric cancer MKN45 cells. (A) Colony formation assay of *H. pylori*-infected MKN45 cells in compared with MKN45 cells alone, and quantitative results of the colony formation numbers. (B) MTT assay of the cell viability of *H. pylori*-infected MKN45 cells in compared with MKN45 cells alone. (C) Invasion and migration assays of *H. pylori*-infected MKN45 cells in compared with MKN45 cells alone. Numbers of invasive and migrated cells were shown as mean ± SD, n = 3. $^{**}p < 0.01$ by Student's t test. All the results were reproducible in three independent experiments.

Figure S2 | Effect of *H. pylori* on the BCL9-TCF/LEF signaling pathway in gastric cancer MKN45 cells. (A) qRT-PCR assay of miR-30a-5p in *H. pylori*-infected MKN45 cells for 12 h and 24 h, normalized to miR-67 expression. (B) qRT-PCR assay for the effect of *H. pylori* on BCL9 mRNA expression in MKN45 cells, normalized to control MKN45 cells. (C) Western blot assay for the effect of *H. pylori* on BCL9 protein expression in MKN45 cells. (D) Luciferase reporter activities assay for the effect of *H. pylori* on the LEF/TCF promoter in MKN45 cells, normalized to pGL3-basic vector. (E) qRT-PCR assay for the effect of *H. pylori* on the transcriptional activity of β-catenin downstream target genes Cyclin D1, MMP7, CD44 and c-Myc in MKN45 cells, normalized to control MKN45 cells. (F) Western blot assay for the effect of *H. pylori* on the protein expression of β-catenin downstream target genes in MKN45 cells. Data are shown as mean ± SD; n = 3. $^{*}p < 0.05$, was considered as statistically significant, $^{**}p < 0.01$, was considered as statistically highly significant.

Figure S3 | Effect of *H. pylori* and miR-30a precursor on COX-2, BCL9 and β-catenin signaling pathway in SGC-7901 cells. (A) qRT-PCR assay for the effect of *H. pylori* on the mRNA expressions of COX-2, BCL9, Cyclin D1 and MMP7 in SGC-7901 cells, normalized to control SGC-7901 cells. (B) Western blot assay for the effect of *H. pylori* on the protein expression of COX-2, BCL9, Cyclin D1 and MMP7 in SGC-7901 cells. (C) Luciferase reporter activities assay for the effect of *H. pylori* on the LEF/TCF promoter in SGC-7901 cells, normalized to pGL3-basic vector. (D) qRT-PCR assay for the effect of miR-30a precursor on the transcriptional levels of COX-2, BCL9, Cyclin D1 and MMP7 in *H. pylori*-infected SGC-7901 cells, in comparing with miR-30a-5p or miR-30a-3p alone, normalized to miR-67 expression. (E) Western blot assay for the effect of miR-30a precursor on the protein expression of COX-2, BCL9, Cyclin D1 and MMP7 in *H. pylori*-infected SGC-7901 cells, in comparing with miR-30a-5p or miR-30a-3p alone. (F) Luciferase reporter activities assay for the effect of miR-30a precursor on the LEF/TCF promoter in *H. pylori*-infected SGC-7901 cells, in comparing with miR-30a-5p or miR-30a-3p alone, normalized to pGL3-basic vector.

Figure S4 | Regulatory effect of *H. pylori* and miR-30a precursor on the growth and migration of gastric cancer SGC-7901 cells. (A) Colony formation assay of *H. pylori*-infected SGC-7901 cells in compared with SGC-7901 cells alone, and quantitative results of the colony formation numbers. (B) Invasion and migration assays of *H. pylori*-infected SGC-7901 cells in compared with SGC-7901 cells alone. (C) Colony formation assay for the effect of miR-30a precursor and miR-30a-5p/ miR-30a-3p inhibitor on the *H. pylori*-infected SGC-7901 cells, and miR-67 mimic was the negative control. (D) Invasion and migration assays for the effect of miR-30a precursor and miR-30a-5p/miR-30a-3p inhibitor on the *H. pylori*-infected SGC-7901 cells, and miR-67 was the control. Numbers of invasive and migrated cells were shown as mean ± SD, n = 3. $^{**}p < 0.01$ by Student's t test. All the results were reproducible in three independent experiments.

Figure S5 | Immunohistochemical and quantitative analysis of COX-2, VEGF and CD34 in mice tissues. (A) Immunohistochemical analysis of COX-2, VEGF, and CD34 proteins on consecutive tissue microarray slides of gastric mucosa from the miR-30a^{KO} mice or WT mice infected by *H. pylori* (scale bars, 200 μm). (B) Quantitative analysis of COX-2 VEGF, and CD34 proteins on consecutive tissue microarray slides of gastric mucosa from the miR-30a^{KO} mice or WT mice infected by *H. pylori*. $^{*}p < 0.05$, was considered as statistically significant, $^{**}p < 0.01$, was considered as statistically highly significant.

Figure S6 | Immunohistochemical analysis of COX-2, BCL9, VEGF and CD34 proteins on consecutive tissue microarray slides of human tissue samples from patients with *H. pylori* gastritis, *H. pylori*-related gastric cancer and healthy controls (scale bars, 100 μm). Normalization to WT mice without *H. pylori* infection.

Figure S7 | Quantitative analysis of COX-2, BCL9, VEGF, CD34 proteins, miR-30a-5p and miR-30a-3p in human tissue samples. (A), (B), (C), (D) Quantitative analysis of COX-2, BCL9, VEGF, and CD34 proteins in human tissue samples from patients with *H. pylori* gastritis, gastric cancer and healthy controls, normalized to healthy controls. (E) Expression detection of miR-30a-5p and miR-30a-3p by qRT-PCR, normalized to healthy controls. $^{*}p < 0.05$, was considered as statistically significant, $^{**}p < 0.01$, was considered as statistically highly significant.

Figure S8 | A schematic model of miR-30a in *H. pylori*-infected gastric cancer. miR-30a inhibited the growth and migration of *H. pylori*-infected gastric cancer cells through double-targeting COX-2 and BCL9, following by affecting β-catenin signaling pathway.
